# The Association Between Threat and Politics Depends on the Type of Threat, the Political Domain, and the Country

**DOI:** 10.1177/0146167220946187

**Published:** 2020-08-26

**Authors:** Mark J. Brandt, Felicity M. Turner-Zwinkels, Beste Karapirinler, Florian Van Leeuwen, Michael Bender, Yvette van Osch, Byron Adams

**Affiliations:** 1 Tilburg University, The Netherlands; 2 Gratia Christian College, Hong Kong, P. R. China; 3 University of Johannesburg, South Africa

**Keywords:** ideology, belief systems, threat, culture

## Abstract

Theories link threat with right-wing political beliefs. We use the World Values Survey (60,378 participants) to explore how six types of threat (e.g., economic, violence, and surveillance) are associated with multiple political beliefs (e.g., cultural, economic, and ideological identification) in 56 countries/territories. Multilevel models with individuals nested in countries revealed that the threat-political belief association depends on the type of threat, the type of political belief, and the country. Economic-related threats tended to be associated with more left-wing economic political beliefs and violence-related threats tended to be associated with more cultural right-wing beliefs, but there were exceptions to this pattern. Additional analyses revealed that the associations between threat and political beliefs were different across countries. However, our analyses identified few country characteristics that could account for these cross-country differences. Our findings revealed that political beliefs and perceptions of threat are linked, but that the relationship is not simple.

The world can be a threatening place. There are wars, pandemics, malicious governments, organized crime syndicates, job losses, educational struggles, food insecurities, natural disasters, and petty crimes. One way that this diverse set of potential threats can be managed is through politics. This includes political and governmental institutions that work to manage threat and mitigate their effects through various political policies (e.g., social welfare) and formal institutions (e.g., the armed forces). In its extreme, this is Hobbes’ *Leviathan* come to life: The state of nature is one of fear and a functioning government can manage that state of nature. Various policies and political beliefs can also help address threat and reduce feelings of fear. People turn to political beliefs that match their personality traits, predispositions, and ongoing epistemic and existential needs (Jost et al., 2009). One class of predispositions includes people’s feelings and experiences of threat (Adorno et al., 1950; Conway et al., 2020; Doty et al., 1991; Duckitt & Sibley, 2009; Lazarev et al., 2014; Wilson, 1973). Adopting specific political beliefs, such as beliefs that are particularly stable and rigid, may help reduce feelings of threat (Thórisdóttir & Jost, 2011).

We examined how different types of threat are associated with different types of political beliefs across a diverse array of countries. In doing so, we not only expand on the types of threat, political beliefs, and countries usually explored in the literature, but we also go beyond this by conducting a joint evaluation of all of these dimensions simultaneously. The results challenge a straightforward relationship between political beliefs and feelings of threat.

## Politics and Threat

The links between political beliefs and feelings of threat form the crux of psychological theories of political ideology (Adorno et al., 1950; Conway et al., 2020; Duckitt & Sibley, 2009; Jost et al., 2003; Onraet, Van Hiel, Dhont, & Pattyn, 2013; Stenner, 2005). The motivated social cognition approach to ideology, for example, highlights existential motivations, especially as they manifest in perceptions and experiences of threat, both as a consequence and as a key predictor of right-wing political beliefs (Jost et al., 2003, 2017).^1^ The idea is that right-wing beliefs help fulfill a variety of basic psychological motivations and needs, including those related to managing uncertainty and fear. Right-wing ideology’s links with the resistance to social change, maintenance of societal traditions, and the acceptance of inequality make it an ideal candidate to help manage fear and feelings of threat, even when the threat is not directly related to politics or specific political issues. As Jost et al. (2017, p. 326) put it, “When one confronts a world that appears dangerous and unpredictable, it is possible to find solace in the maintenance of what is familiar and known (the status quo) and entrust one’s fate to powerful, prestigious authority figures.” In other words, people adopt political beliefs that help them fulfill particular psychological motivations, and right-wing ideology may be particularly well suited to help people manage threat.

Consistent with the motivated social cognition approach, a recent meta-analysis across multiple measures of fear and existential threat finds that the association between threat and right-wing beliefs was small-to-moderate (depending on the precise measure; Jost et al., 2017; see also Onraet, Van Hiel, Dhont, & Pattyn, 2013). Other work (not summarized in the meta-analysis), has linked right-wing political beliefs with stronger physiological reactions to negative and threatening stimuli and a general negativity bias (for a review see Hibbing et al., 2014; but see Bakker et al., 2020; Osmundsen et al., in press for reasons to doubt such physiological effects). On the basis of this literature (e.g., Jost et al., 2017), one might expect that the link between threat and right-wing beliefs is robust.

## Expanding Stimuli and Samples

We suggest that a broad and general link between threat and politics is too simple. Although there are likely specific conditions under which there is a robust link between threat and right-wing political beliefs, we suspect that when broader samples of (a) types of political beliefs, (b) types of threat, and (c) populations of participants are taken into account the link will be more fragile. In our work, we not only explore the relation between ideology and threat, but move beyond this to examine the link between different types of threat and different types of political beliefs among participants from a large sample of countries. Prior work has examined different types of threat and different types of beliefs. We build on this work by expanding the types of threat and examining the joint influence of the types of threat, types of political beliefs, and countries simultaneously. This helps identify the variability in the link between threat and politics across these three factors. This is consistent with calls for using broader and more representative arrays of stimuli and political systems in political psychology (Baron & Jost, 2019; Brandt & Crawford, 2019; Brandt & Wagemans, 2017; Kessler et al., 2015) and psychology more broadly (Henrich et al., 2010). When researchers focus on a narrow range of stimuli or samples, heterogeneity between stimuli or samples is often missed and the size and consistency of effects are likely to be overestimated.

## Multiple Dimensions of Political Beliefs

The first way we expand our test of the link between threat and politics is by considering cultural and economic dimensions of political beliefs (Choma & Hodson, 2017). Although some scholars highlight a tight correspondence between cultural and economic dimensions (Azevedo et al., 2019), there is a consensus that political beliefs can be split into correlated, but theoretically independent dimensions that are related to cultural political beliefs (including the maintenance of traditions and national boundaries) and economic policies and beliefs (including the maintenance of inequality; Crawford et al., 2017; Everett, 2013; Feldman & Johnston, 2014; Johnston et al., 2017; Johnston & Ollerenshaw, 2020; Malka et al., 2019; Nilsson et al., 2019). Cultural and economic dimensions of political beliefs may fulfill different psychological functions and so may have different relationships with feelings of threat.

For example, right-wing beliefs in the cultural domain may be especially adept at addressing feelings of threat because of their close connection with the resistance to social change and social traditions. In contrast, right-wing economic political beliefs may be less capable of addressing threat because they do not provide the same levels of certainty (e.g., economic markets can be uncertain). Instead, left-wing economic political beliefs may be better suited to address feelings of threat because the policies associated with these left-wing economic political beliefs tend to provide more certainty (e.g., via unemployment support). The idea is that because of the certainty embedded in right-wing cultural political beliefs *and* left-wing economic political beliefs, both beliefs are especially likely to help address people’s perceptions and general feelings of threat. This line of thinking suggests that threat will be related to more *right-wing cultural political beliefs* and more *left-wing economic political beliefs*. And this is what has been found (Feldman & Johnston, 2014; Johnston et al., 2017; Malka et al., 2014, 2019; van Hiel et al., 2004). Our work directly builds on this prior work by investigating how multiple dimensions of political beliefs are related to multiple types of threat across multiple political systems.

## Multiple Types of Threat

The second way we expand our test between threat and politics is by considering dimensions of threat (Crawford, 2017). Although some people may experience generalized feelings of threat (Onraet, Van Hiel, Dhont, & Pattyn, 2013), oftentimes threat is experienced toward a specific target (e.g., Stephan & Renfro, 2002). For example, a person who has experienced a job loss may be more prone to feelings of economic threat, but not toward the threat of potential crime. Conversely, a person may be quite threatened by general criminal activity and have stable employment. To the extent that particular political beliefs are better at addressing some types of threat compared with others, the relationship between threat and politics should depend on the type of threat.

There is research consistent with this idea. For example, Jost and colleagues (2017) summarize data from a representative sample of the United States where participants were asked about their fear toward 73 different stimuli and also reported their political ideology. They found that rightists tended to report more fear of gun control, illegal immigration, and governmental corruption, whereas leftists tended to report more fear of climate change, pollution, and overpopulation. Experimental studies, also conducted in the United States, have shown that threats of restricted health-care access, pollution, and corporate misconduct all increase support for leftist beliefs in the threatened domain (e.g., more leftist health-care beliefs following the threat to health-care access; Eadeh & Chang, 2019; see also Fiagbenu et al., in press). Other work finds that threat to society (broadly) increase right-wing authoritarianism, whereas the threat of a particularly competitive environment is associated with increased social dominance orientation (for a review Duckitt & Sibley, 2009). A common thread between the work by Eadeh and Chang (2019) and Duckitt and Sibley (2009) is that people adopt political beliefs that best address the threat they are experiencing. If right-wing beliefs are better able to address some threats (e.g., from violence) compared with others (e.g., from unemployment), then different threats will likely have different relationships with right-wing ideology. In particular, the association is likely to be stronger for threat that can be addressed by right-wing beliefs, but maybe negligible for threats that are beyond the reach of right-wing beliefs. A shortcoming of this prior work is that it primarily focuses on one particular political system (e.g., the United States, as in Eadeh & Chang, 2019). Our work builds on this prior work by expanding the types of threats considered and then assessing if these threat-politics associations are consistent across multiple political systems.

## Multiple Political Systems

A third way to expand tests of the link between threat and politics is by considering political beliefs within different countries and political systems. Political beliefs are not timeless and stateless, but rather are situated in different histories, institutions, and cultures, which all may affect how the beliefs develop, are expressed, and address feelings of threat. Much of the work on threat and politics is from the United States, with some also coming from Western Europe. For example, only ~4% of the data from Jost and colleagues’ (2003) meta-analysis comes from outside of Western Europe, North America, Australia, and New Zealand, mirroring a common issue in psychological research (Henrich et al., 2010). More data were from the United States than from all of the other countries combined (~75%). This makes it near impossible to understand if and to what extent the associations between threats and political beliefs differ across countries.

There is research that assessed the link between various existential motivations and political beliefs. Some of this work is conducted at the country-level or the state-level of analysis. For example, in one project indicators of threat at the country-level (e.g., poor unemployment, low GDP per capita) were associated with right-wing beliefs at the country-level (Onraet, van Hiel, & Cornelis, 2013; see also Conway et al., 2017). However, it currently remains unclear how such country-level results relate to individuals’ endorsement of threat/political belief. Indeed, work that focuses on the country-level, state-level, or any aggregate level does not necessarily generalize to the individual- and psychological-level of analysis (see discussions of the ecological fallacy or cross-cultural isomorphisms; e.g., Robinson, 1950; Van de Vijver et al., 2008). For example, cultural and economic political beliefs appear to be positively correlated at the country-level of analysis (Onraet, van Hiel, & Cornelis, 2013), yet using similar data these beliefs appear to be negatively correlated in many countries (Malka et al., 2019). This means that findings at the country-level are conceptually different and do not necessarily inform the psychological theories we are building on in this project.

This is not to say that the link between country characteristics and individual-level political beliefs has not been explored at all. There are projects that have assessed how values of security are associated with political beliefs across a range of countries. In these projects, the link between security values and political beliefs is estimated at the individual-level of analysis. Additional analyses then test if this individual-level association is similar or different across countries by testing whether characteristics of the country moderate the individual-level association between security values and political beliefs. That is, these projects test if people’s political psychology differs across countries. For example, in an analysis among Europeans, valuing security was associated with more right-wing identification in Western Europe, but more left-wing identification in Eastern Europe (Thorisdottir et al., 2007). This effect was later also confirmed outside of Europe (Malka et al., 2014). Analyses using this broader sample found that values of order and security are positively associated with right-wing cultural political beliefs, but that this association is largest among individuals in countries with high levels of human development and ideological constraint (i.e., countries where cultural and economic political beliefs are more highly intercorrelated; Malka et al., 2014). In contrast, values of order and security tend to be associated with left-wing economic political beliefs and this association is smaller in ideologically constrained and non-Eastern European countries. Taken together, these results suggest that the link between valuing order and security and political ideology at the individual-level depends on the type of political beliefs and characteristics of the country. A clear downside of these studies for our purposes is that they assess people’s *values* using Schwartz’s Portrait Values Questionnaire (Schwartz et al., 2001), thus leaving it unclear if these patterns apply to the association between *threat* and political beliefs.

## Country Characteristics

We sought to *explore* a wide range of country characteristics that might help us understand when threat is associated with particular political beliefs. Although some past studies have looked at country characteristics like the Human Development Index (HDI), location in Eastern Europe, ideological constraint, or traditionalism (Malka et al., 2014, 2019; Thorisdottir et al., 2007), this is a limited range of possible country characteristics. Countries can differ in a number of different ways not captured so far, including the quality of their institutions, the primary drivers of their economy, the levels of inequality, the levels of individualism/collectivism, and more. Given the broad range of ways countries can differ, we adopted a broad and purposefully exploratory approach (Jebb et al., 2017) that examined 26 country characteristics drawn from political science, sociology, and cross-cultural psychology. This allows us to take an inductive approach that pushes our knowledge of where the link between threat and political beliefs is more or less likely to emerge.

The specific country characteristics and the rationale for including them are in Table 1. Some of these characteristics tap into conceptually similar things (e.g., quality of government) and so the rationale for such measures are included just once. We chose characteristics that have been mentioned in the literature (e.g., Malka et al., 2014, 2019; Thorisdottir et al., 2007), but also sought to expand the range of typical characteristics studied in this domain. That means that our rationale for including such measures ranges from tests of theoretical ideas posited in related literature (e.g., quality of government, HDI, ideological constraint) to much more exploratory rationales and curiosity. Predictions for country characteristics with firmer theoretical foundations are highlighted in gray in Table 1. We also elaborate more on these characteristics in the results.

**Table 1.** Country Characteristics and Rationale for Inclusion in the Study.

Country characteristic (source)	Description	Rationale
Corruption Perception Index^a^ (Norris, 2015)	Perceived levels of public sector corruption based on experts and opinion surveys	Threat may be more strongly associated with political beliefs that give governments power (e.g., economic left-wing beliefs) when a country has a well-functioning government (Kay et al., 2008).
Government Effectiveness Index (Kaufmann & Kraay, 2019)	Perceptions and quality of public services	
Governance Quality (Norris, 2015)	Expert ratings of the quality of governance.	
Democratic Governance Index (Norris, 2015)	A measure combining the extent of liberal democracy and the quality of government	
Former member of Eastern bloc	Was the country a part of the USSR?	Threat may be more likely to be associated with left-wing economic political beliefs when the status quo is more left-wing on the economic dimension because threat motivates people to maintain the status quo (Thorisdottir et al., 2007).
Trust Index	Proportion of people in the country reporting that “most people can be trusted.”	People may be less likely to turn to strangers, including the government, for help in countries with low levels of trust (Bergh & Bjørnskov, 2011). This may mean that the link between threat and left-wing economic political beliefs are less likely in low trust countries.
Human Development Index (United Nations, 2017)	Composite of life expectancy, education, and per capita income	Threat may be less associated with right-wing political views when economic conditions are poor because the economically threatening context makes everyone more right-wing (Sibley et al., 2012).
Gini Index (World Bank, 2019d)	Extent of income inequality	
Gender Inequality Index (United Nations, 2017)	Composite of reproductive health and gender inequality in political, education, and the labor market	
Diversity Index, language (Forbes, 2012)	Employee diversity based on language	Threat may be more strongly associated with right-wing political beliefs when diversity attributed to immigration and multiculturalism is high (e.g., because it is a symbolic threat to the national identity; Morrison & Ybarra, 2009; Smeekes & Verkuyten, 2014).
Diversity Index, country of birth (Forbes, 2012)	Employee diversity based on country of birth	
Linguistic Diversity Index (UNESCO, 2009)	The probability that any two people selected would have different mother tongues	
International Migrant Stock (United Nations, 2017)	Number of migrants in a country at a given time.	
Freedom of Expression and Belief Index (Freedom House, 2016)	Expert ratings of cultural, religious, and academic freedom	Threat may be more weakly associated with right-wing cultural political beliefs in countries with homogenous, strong religious norms because those norms make everyone more right-wing on cultural issues (Hoffarth et al., 2018).
Religious Freedom Index (Norris, 2015)	Composite measure of the state’s involvement in the regulation of religion	
Religious Diversity Index (Johnson & Grim, 2018)	Extent one religion is prevalent or if there are many religions	
Importance of Religion (Crabtree, 2010)	Percent indicating that religion is an important part of daily life	
KOF Globalization Index (Gygli et al., 2019)	Composite of indicators that the country is politically and economically connected internationally	Lower levels of globalization are related to higher levels of nationalism (Ariely, 2012). This may weaken the link between threat and right-wing beliefs because lower levels of globalization would make everyone more right-wing.
Individualism/collectivism (Hofstede, 2015)	Extent people from the country express individualistic versus collectivistic values	The relative individualism or collectivism norms in a country may affect the relationship between threat and political beliefs because it may be related to different norms for addressing threat (Jetten et al., 2002).
Individualism/collectivism (Minkov, 2018)	Extent people from the country express individualistic versus collectivistic values	
Tightness Index (Gelfand et al., 2011)	Extent a country has strong norms and low tolerance for deviant behaviors.	Threat may be more weakly associated with right-wing cultural political beliefs in tight countries than in loose countries, because of the strength of norms (norms are clear and pervasive in tight cultures) and the strength of sanctioning (there is less tolerance for normative deviance in tight cultures; Gelfand et al., 2006).
Percent agriculture (World Bank, 2019a)	Percent of total employment in agriculture	Mode of subsistence seems to influence how people socialize their children which may affect political expression. For example, in societies with more agriculture, people socialize their children more toward compliance (Kağitçibaşi, 2007) and in societies with more service jobs, people might be socialized to more expression of emotions (Van Hemert et al., 2007).
Percent industry (World Bank, 2019b)	Percent of total employment in industry	
Percent service (World Bank, 2019c)	Percent of total employment in service	
Climate Index^b^ (Van de Vliert, 2007)	Extent climate deviates from 22°C	Climate helps define constraints and affordances for specific cultural adaptations to the environment (Van de Vliert, 2007), such as agricultural techniques and self/other orientation (see Talhelm et al., 2014). Such cultural adaptions may be relevant for how people respond to threat. Prior work has linked it with harsh governance (Van de Vliert & Tol, 2014).
Ideological Constraint	Average inter-item correlation of the political items in the study.	Threat may be more likely to be associated with right-wing economic political beliefs in a country with ideological constraint because they are more likely to be exposed to political discourse that constrains political attitudes on a single right-left dimension (Malka et al., 2014).

a Higher scores indicate less corruption. ^b^ Average temperatures were obtained from weatherbase.com. Index is calculated according to the following formula: (22-average hottest)^2^ + (22-average lowest)^2^. Predictions for country characteristics with firmer theoretical foundations are highlighted with gray.

## The Current Study

The purpose of the current study is to explore how threat is associated with political beliefs when expanding on the types of threat, types of political beliefs, and countries under study. Although prior work has looked at these different domains, our key contribution is examining all of these different domains in the same study. This allows for a direct assessment of the extent stimuli variation in types of threat, types of political beliefs, and countries under study affect our understanding of the association between threat and political beliefs. Focusing on one type of threat, political belief, or country could give a misleading impression about the consistency of the associations between threat and polices.

We use data from the 6th wave of the World Values Survey (Inglehart et al., 2014) which includes measures of threats related to violence (e.g., war), neighborhoods (e.g., lack of security and crime in one’s neighborhood), the police (e.g., interference of the police and racism), economics (e.g., unemployment, lack of education), poverty (e.g., lack of cash, food, and medicine), and government surveillance.^2^ The threats tap into several of the threats previously studied (e.g., economic, violence, and social threats; Doty et al., 1991; Huddy et al., 2005; Onraet, Van Hiel, & Cornelis, 2013; Onraet, Van Hiel, Dhont, & Pattyn, 2013), as well as threats less commonly considered (e.g., surveillance and police threats). Although our study is exploratory, based on our reading of the literature, and the idea that people will adopt the political beliefs that are perceived as best addressing a particular threat (Duckitt & Sibley, 2009; Eadeh & Chang, 2019), we formed four expectations:

1. We expect that threats related to economics and poverty would be associated with more left-wing economic political beliefs, because left-wing economic political beliefs are typically perceived to address threat in the domains of social security, unemployment, health, and the environment across countries (Seeberg, 2017).
2. We expect that threats related to violence and an insecure neighborhood would be associated with more right-wing cultural political beliefs because right-wing cultural political beliefs are typically perceived to address threats to law and social order across countries (Seeberg, 2017).
3. We expect that threats related to the police would be associated with more left-wing beliefs in the cultural domain because right-wing cultural political beliefs are often associated with law and social order across countries (Seeberg, 2017), which is not likely to help address the ongoing threat of the police.
4. We expect that the association between threat and politics will be moderated by the quality of the government, the countries’ history as a member of the Eastern Bloc, the economic conditions, and the ideological constraint in the country (see Table 1).

We do not have any expectations for how the threat of government surveillance will be associated with political beliefs, in part because issues related to surveillance are not asked in issue ownership studies (Seeberg, 2017) and this threat is understudied more generally. Nonetheless, this type of threat is important to study due to its centrality in an increasingly surveilled world and its centrality to some political ideologies (e.g., libertarianism) and conspiracy theories. Although the threat of government surveillance was associated with right-wing beliefs in the United States (e.g., Jost et al., 2017), it is not obvious that this would generalize to other political contexts.

Although our expectations for the link between threat and policies were expressed in terms of cultural and economic political beliefs, we also include a measure of ideological identification. This measure is one of the most often used measures to test social psychological hypotheses about political beliefs (e.g., Jost, 2006), including those regarding threat (e.g., Jost et al., 2017). It helps address the basic claim made by some that right-wing beliefs are a general antidote to threat (e.g., Hibbing et al., 2014). Past work finds more similarities between ideological identification and cultural political beliefs than economic political beliefs (Malka et al., 2014), suggesting that the associations between threat and ideological identification will be similar to the associations between threat and cultural political beliefs. In short, our study helped us test if the association between threat and politics depends on the type of threat, the political domain, and the country when simultaneously assessed.

## Method

### Participants and Procedure

We used data from the 6th wave of the World Values Survey (1981–2014) which includes representative samples from around the world collected between 2010 and 2014. After excluding participants who did not complete the threat measures and at least one of the political measures, our analyses included data from 60,378 participants (49% men, 51% women, 0.0003% missing gender data, mean age = 41.5 years, *SD* = 16.1) from 56 countries (mean *N*/country = 1,078, *SD* = 456). We have 80% power to detect a correlation of .09 in a country with the average sample size. Table S1 in the Supplemental Material includes a complete list of country sample sizes. We have 80% power to detect a correlation of .13 in the country with the smallest sample size (Turkey, *n* = 481).

### Measures

#### Political beliefs

The data contained seven political beliefs related to the economic domain and the cultural domain, as well as people’s ideological self-identification as left-wing or right-wing (see Table 2). All of the items are the same as the items used by Malka and colleagues’ (2019) work on the structure of political beliefs across countries, with one exception. Malka and colleagues (2019) used an item asking participants about their views of immigration on a scale ranging from “Let anyone come who wants to” to “Prohibit people coming here from other countries.” However, this item was only available in a limited number of countries. To maximize coverage, we chose an item about immigration that was available in more countries. All political items were coded such that higher scores indicated more right-wing positions and lower scores indicated more left-wing positions on those issues.

**Table 2.** Political Belief Items.

Economic political beliefs
Inequality is okay: *M r* = .25, *SD r* = .15, range *r* = [−.07, .56]
1 = *incomes should be made more equal*, 10 = *we need larger income differences as incentives for individual effort*
1 = *people should take more responsibility to provide for themselves*, 10 = *the government should take more responsibility to ensure that everyone is provided for*; reverse scored
Less government ownership:
1 = *private ownership of business and industry should be increased*, 10 = *government ownership of business and industry should be increased*; reverse scored
Cultural political beliefs
Social conservatism: *M r* = .46, *SD r* = .14, range *r* = [.15, .73]
Homosexuality (1 = *never justifiable*, 10 = *always justifiable*); reverse scored
Abortion (1 = *never justifiable*, 10 = *always justifiable*); reverse scored
Jobs for high status: *M r* = .20, *SD r* = .10, range *r* = [−.02, .40]
When jobs are scarce, men should have more right to a job than women (1 = *disagree*, 2 = *neither*, 3 = *agree*)
When jobs are scarce, employers should give priority to people of this country over immigrants (1 = *disagree*, 2 = *neither*, 3 = *agree*)
Ideological identification
In political matters, people talk of “the left” and “the right.” How would you place your views on this scale, generally speaking?” (1 = *left*, 10 = *right*).

We used exploratory principle axis factor analysis with the country-mean centered variables (to remove between-country variance) to reduce the number of political items (full description and the correlation matrix used for the factor analysis is in the Supplemental Material). We identified five components. Three are theoretically sensible factors with multiple items. Two items did not load well on any of the factors and we treated these items as separate measures. Although the items about jobs for men and immigrants have some economic content (i.e., jobs), we classified them conceptually as a cultural belief because these items appear to be about protecting traditional societal structures (gender roles) and the cultural ingroup. See Table 2 for the items, their division into scales, and the mean, standard deviation, and range of the scales’ reliability across countries. All multi-item scales were constructed by averaging together the items.

#### Threat

Fourteen items were available to measure threat (see Table 3). These items are similar to items used to measure threat in past work (e.g., Huddy et al., 2005), with the addition of items about surveillance and police-related threats. We used exploratory principle axis factor analyses with the country-mean centered variables (to remove between-country variance) to reduce the number of threat items (full description and the correlation matrix used for the factor analyses is in the Supplemental Material). We identified five theoretically sensible factors, with the addition of the surveillance threat item assessing a sixth threat. See Table 3 for the items, their division into scales, and the mean, standard deviation, and range of the scales’ reliability across countries. All multi-item scales were constructed by averaging together the items.

**Table 3.** Threat Items.

Violence threat: *M* α = .89, *SD* α = .08, range α = [.43, .97]
To what degree are you worried about the following situations . . . A terrorist attack (1 = *not at all*, 2 = *not much*, 3 = *a great deal*, 4 = *very much*)
To what degree are you worried about the following situations . . . A civil war (1 = *not at all*, 2 = *not much*, 3 = *a great deal*, 4 = *very much*)
To what degree are you worried about the following situations . . . A war involving my country (1 = *not at all*, 2 = *not much*, 3 = *a great deal*, 4 = *very much*)
Neighborhood threat: *M* α = .55, *SD* α = .12, range α = [.15, .75]
In the last 12 months, how often have you or your family . . . Felt unsafe from crime in your home (1 = *often*, 2 = *sometimes*, 3 = *rarely*, 4 = *never*); reverse scored
How frequently do the following things occur in your neighborhood? . . . Robberies (1 = *very frequently*, 2 = *quite frequently*, 3 = *not frequently*, 4 = *not at all frequently*); reverse scored
Could you tell me how secure do you feel these days in your neighborhood? (1 = *very secure*, 2 = *quite secure*, 3 = *not very secure*, 4 = *not at all secure*)
Police threat: *M r* = .48, *SD r* = .13, range *r* = [.20, .76]
How frequently do the following things occur in your neighborhood? . . . Police or military interfere with people’s private life (1 = *very frequently*, 2 = *quite frequently*, 3 = *not frequently*, 4 = *not at all frequently*); reverse scored
How frequently do the following things occur in your neighborhood? . . . Racist behavior (1 = *very frequently*, 2 = *quite frequently*, 3 = *not frequently*, 4 = *not at all frequently*); reverse scored
Economic threat: *M r* = .54, *SD r* = .11, range *r* = [.24, .71]
To what degree are you worried about the following situations . . . Not being able to give my children a good education (1 = *not at all*, 2 = *not much*, 3 = *a great deal*, 4 = *very much*)
To what degree are you worried about the following situations . . . Losing my job or not finding a job (1 = *not at all*, 2 = *not much*, 3 = *a great deal*, 4 = *very much*)
Poverty threat: *M* α = .76, *SD* α = .08, range α = [.51, .91]
In the last 12 months, how often have you or your family . . . Gone without medicine or medical treatment that you needed (1 = *often*, 2 = *sometimes*, 3 = *rarely*, 4 = *never*); reverse scored
In the last 12 months, how often have you or your family . . . Gone without enough food to eat (1 = *often*, 2 = *sometimes*, 3 = *rarely*, 4 = *never*); reverse scored
In the last 12 months, how often have you or your family . . . Gone without a cash income (1 = *often*, 2 = *sometimes*, 3 = *rarely*, 4 = *never*); reverse scored
Surveillance threat:
To what degree are you worried about the following situations . . . Government wire-tapping or reading my mail or email (1 = *not at all*, 2 = *not much*, 3 = *a great deal*, 4 = *very much*)

#### Country characteristics

The country-level variables that we used are listed in Table 1 with their relevant references. When multiple years of data were available, we chose the value from the year of data collection of the World Value Survey (WVS) for that particular country. If this year was not available, we chose the value from the closest year (the most common deviation was 0 years). To ensure that any effects of country characteristics were not due to broad regional similarities (e.g., Kuppens & Pollet, 2014, 2015; Pollet et al., 2014; Ross & Homer, 1976), we used region as a sum-to-zero contrast coded covariate (e.g., coding Europe, Sub-Saharan Africa, etc.). To render coefficients of all of the country characteristics comparable, we standardized all of the country characteristics to range from 0 to 1. Correlations between the country-level characteristics are available in the Supplemental Material. Data were not available for all countries. Therefore the country-level *N* ranged from 19 (cultural tightness) to 56 (location in Eastern Europe, religious diversity; median country-level *N* = 55).

## Results

The analysis proceeded in three steps. First, we estimated the average associations between the six threats and the five political beliefs across countries. Second, we assessed whether there was significant between-country variability in the associations between each threat and each political belief. Third, we tested if each of the country characteristics moderated the link between threat and political beliefs.

### Overall Associations Between Threat and Politics

**Figure 1.**
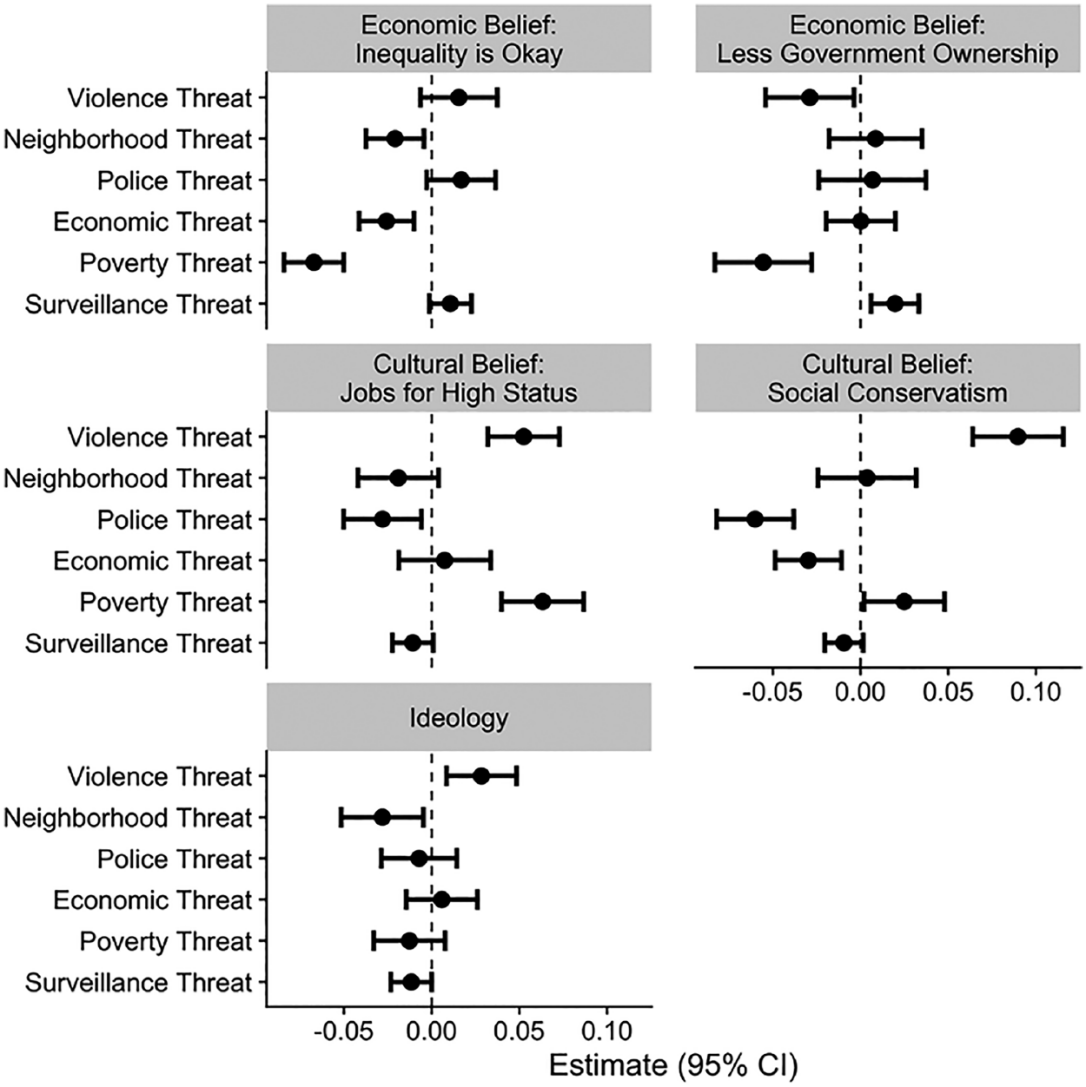
Estimates of the associations and 95% confidence intervals between threat and political beliefs.

To estimate the overall associations between threat and politics, we regressed each of the five political belief measures on the six threat measures in multilevel models with random intercepts and random slopes for the countries (five multilevel regressions in total). All measures were rescaled to range from 0 to 1; this means that the coefficients can be interpreted as the proportion difference of the outcome variable between the minimum and maximum of the predictor variable (e.g., an effect of .10 is a 10% difference in the political variable between the minimum and maximum of the threat variable). The threat measures were country-mean centered. The models were estimated using lme4 (Bates et al., 2015) and lmerTest (Kuznetsova et al., 2017) in *R* (R Core Team, 2019). Figures were created using ggplot2 (Wickham, 2016), cowplot (Wilke, 2019), and ggrepel (Slowikowski, 2019). The coefficient estimates and their confidence intervals are in Figure 1.

Our first expectation was that economically relevant threats (i.e., economic and poverty threats) would be associated with more left-wing economic political beliefs. This would be supported by negative associations between economically relevant threats and the economic political beliefs (inequality is okay and less government ownership). Some support was found for this expectation (Figure 1, Top Row). There were negative associations between both economic and poverty threat and belief that inequality is okay. There was also a negative association between poverty threat and the belief that there should be less government ownership. However, economic threat was not associated with the belief that there should be less government ownership.

Our second expectation was that threats related to violence (i.e., violence and neighborhood threats) would be associated with more right-wing cultural political beliefs. There was some support for this expectation (Figure 1, Middle Row): Violence threats related positively to both measures of cultural political beliefs. Neighborhood threats, however, were not related to either measure of cultural political beliefs. Consistent with the idea that ideological identification is more likely to track cultural political beliefs, violence threats were associated with more right-wing identification; however, unexpectedly, neighborhood threats were associated with more left-wing identification.

Our third expectation was that threats related to the police would be associated with more left-wing cultural political beliefs. As expected, experiencing police threats was associated with more left-wing cultural political beliefs on both indicators of cultural political beliefs (Figure 1, Middle Row).

Notably, there were additional significant associations between the threats and political beliefs that are not captured by our expectations. For example, violence threat was associated with more left-wing views on government ownership, neighborhood threat was associated with more left-wing views on inequality, economic threat was associated with more left-wing views on social conservatism, poverty threat was associated with more right-wing views on both jobs for high status and social conservatism, and surveillance threat was associated with more right-wing views on government ownership. In total, there were six significant association between right-wing beliefs and threat, nine significant associations between left-wing beliefs and threat, and 15 estimates that were not significantly different from zero. The idea that more threat is associated with more right-wing beliefs does not hold when considering multiple threats and multiple political beliefs.

### Variation in Threat-Politics Association Between Countries

The results of the overall analysis are informative, but it averages across any between-country variation. To test if there is significant variation in the associations across country, we tested the significance of the random slopes. To do this, we first estimated the same models as presented in Figure 1, but without any random slopes (only a random intercept). Then, we estimated a model that included the random slope for one of the six threats. Finally, we compared the fit of the two models (no random slopes vs. one random slope) using the ANOVA function in R to assess if each individual slope had significant variation across countries. In all cases, the model including the random slope fit the data better (all *p*s < .001) indicating significant variation. We can also compare the model with all random slopes (i.e., the model in Figure 1) to the model without any random slopes. The model with all random slopes also fit the data better than the model without any random slopes (*p* < .001). Across all threat-politics associations, there is significant variation across countries.

**Figure 2.**
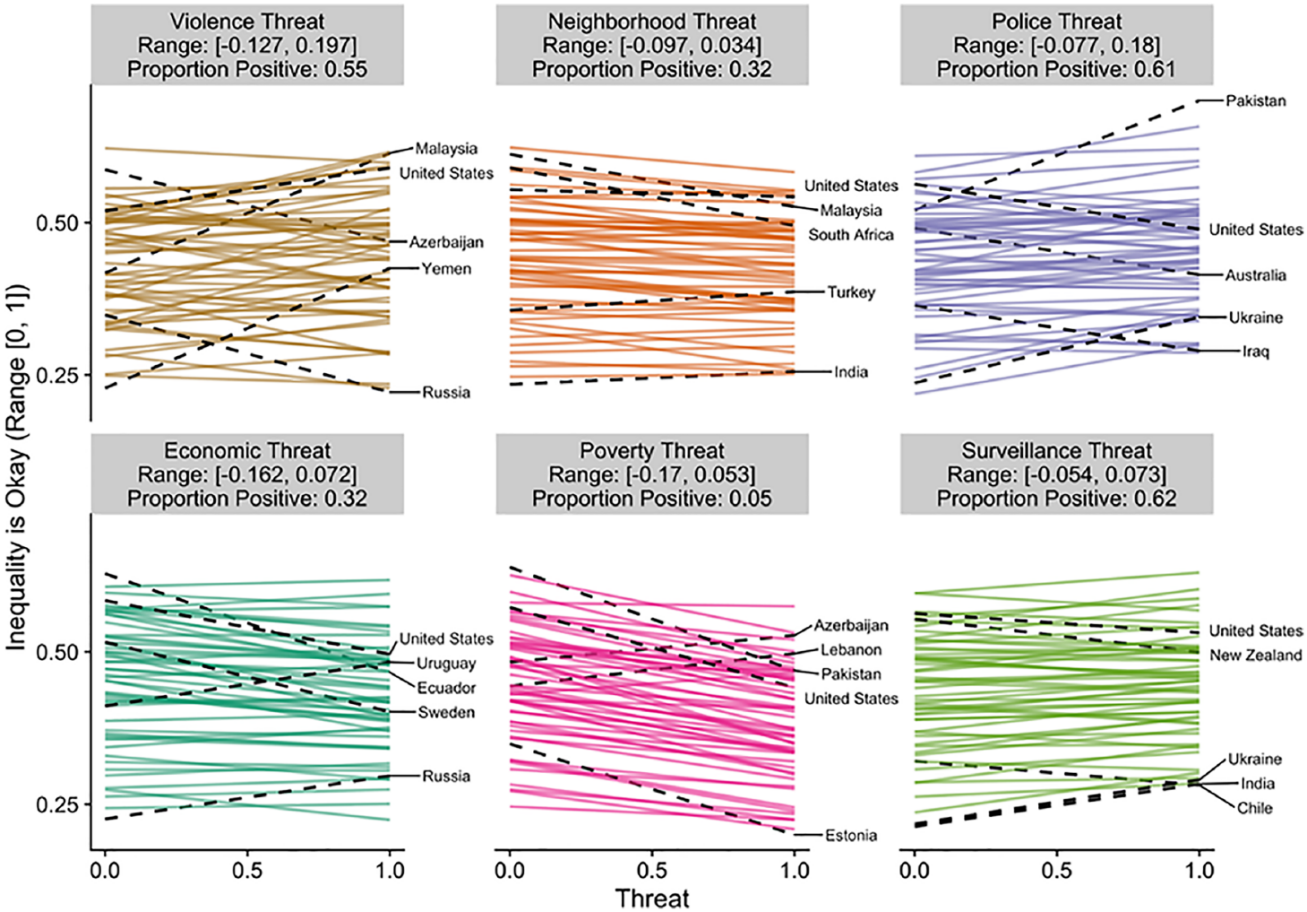
Estimated slopes between threat and inequality is okay for each country. *Note.* The two largest and two smallest slopes are highlighted and labeled. The United States is also highlighted and labeled for comparison purposes. See also Table S5.

**Figure 3.**
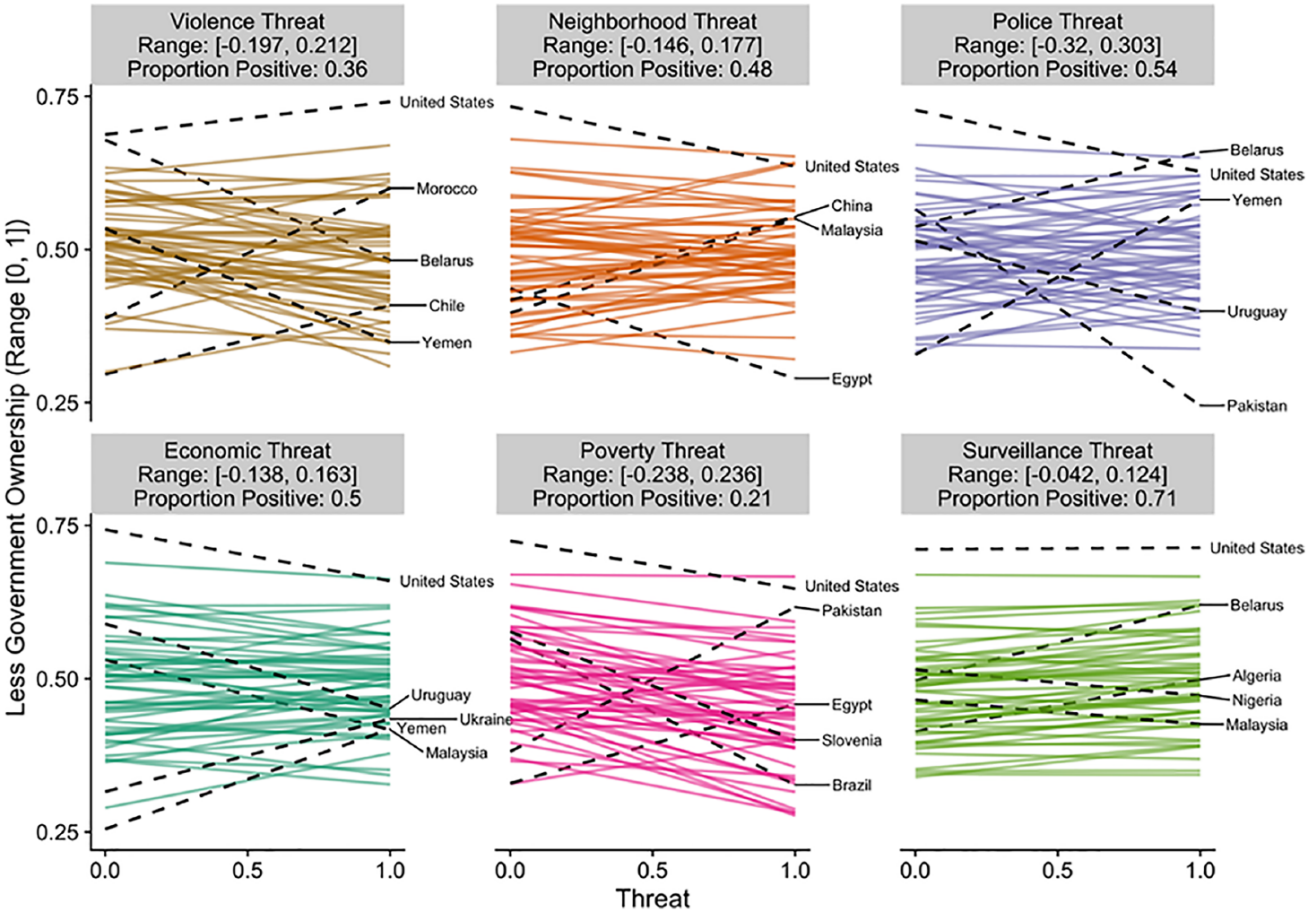
Estimated slopes between threat and less government ownership for each country. *Note.* The two largest and two smallest slopes are highlighted and labeled. The United States is also highlighted and labeled for comparison purposes. See also Table S6.

**Figure 4.**
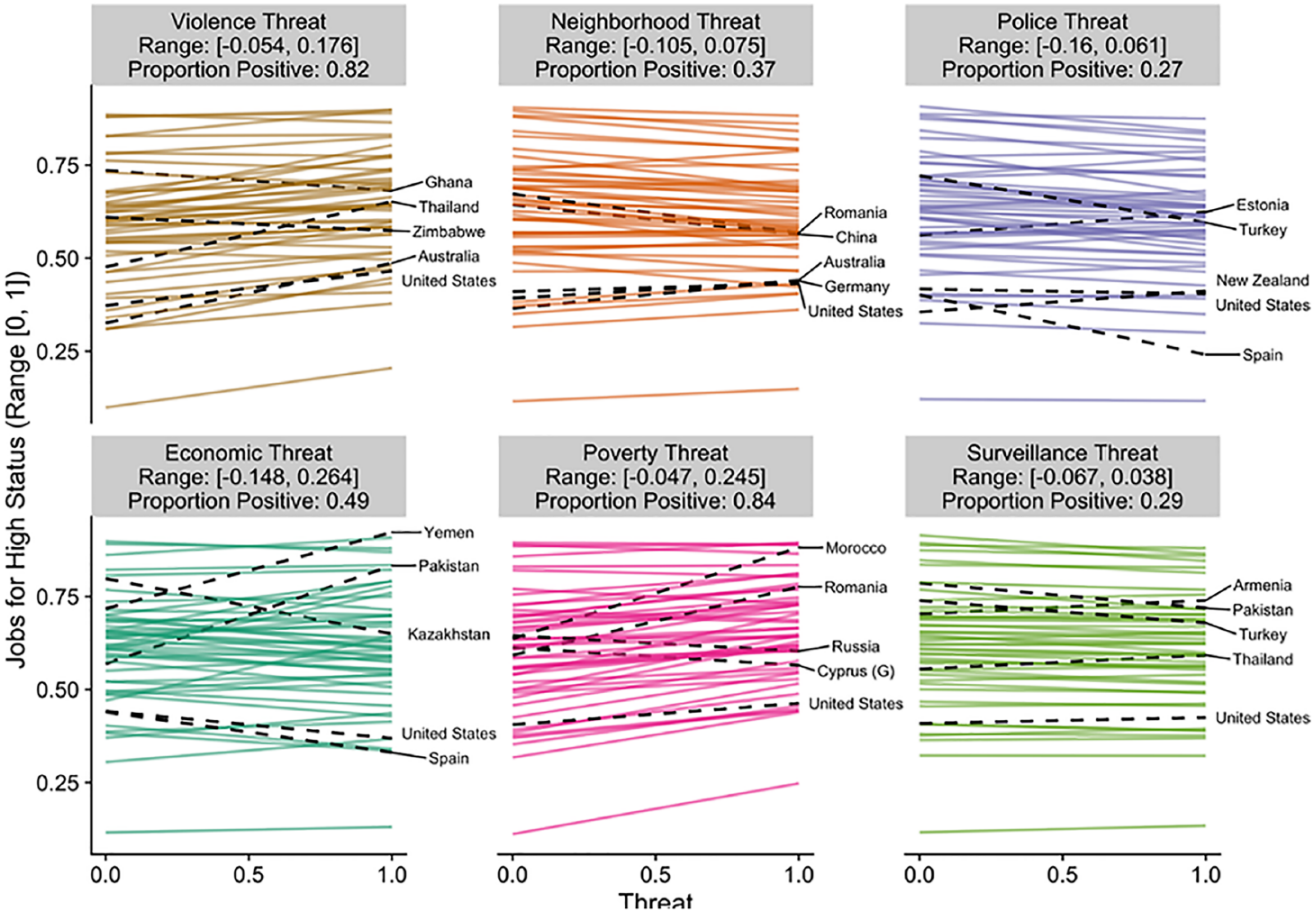
Estimated slopes between threat and jobs for high status for each country. *Note.* The two largest and two smallest slopes are highlighted and labeled. The United States is also highlighted and labeled for comparison purposes. See also Table S7.

**Figure 5.**
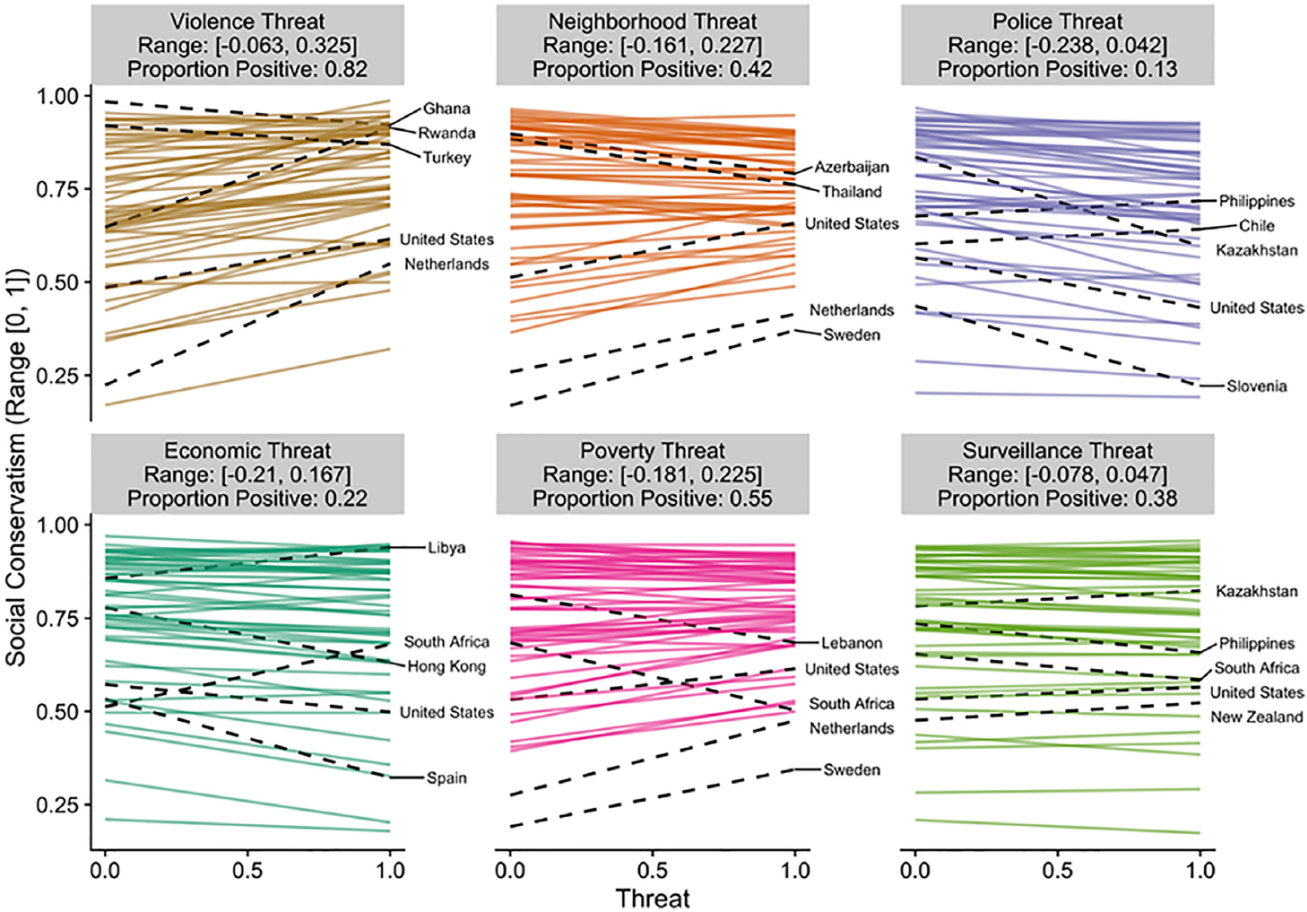
Estimated slopes between threat and social conservatism for each country. *Note.* The two largest and two smallest slopes are highlighted and labeled. The United States is also highlighted and labeled for comparison purposes. See also Table S8.

**Figure 6.**
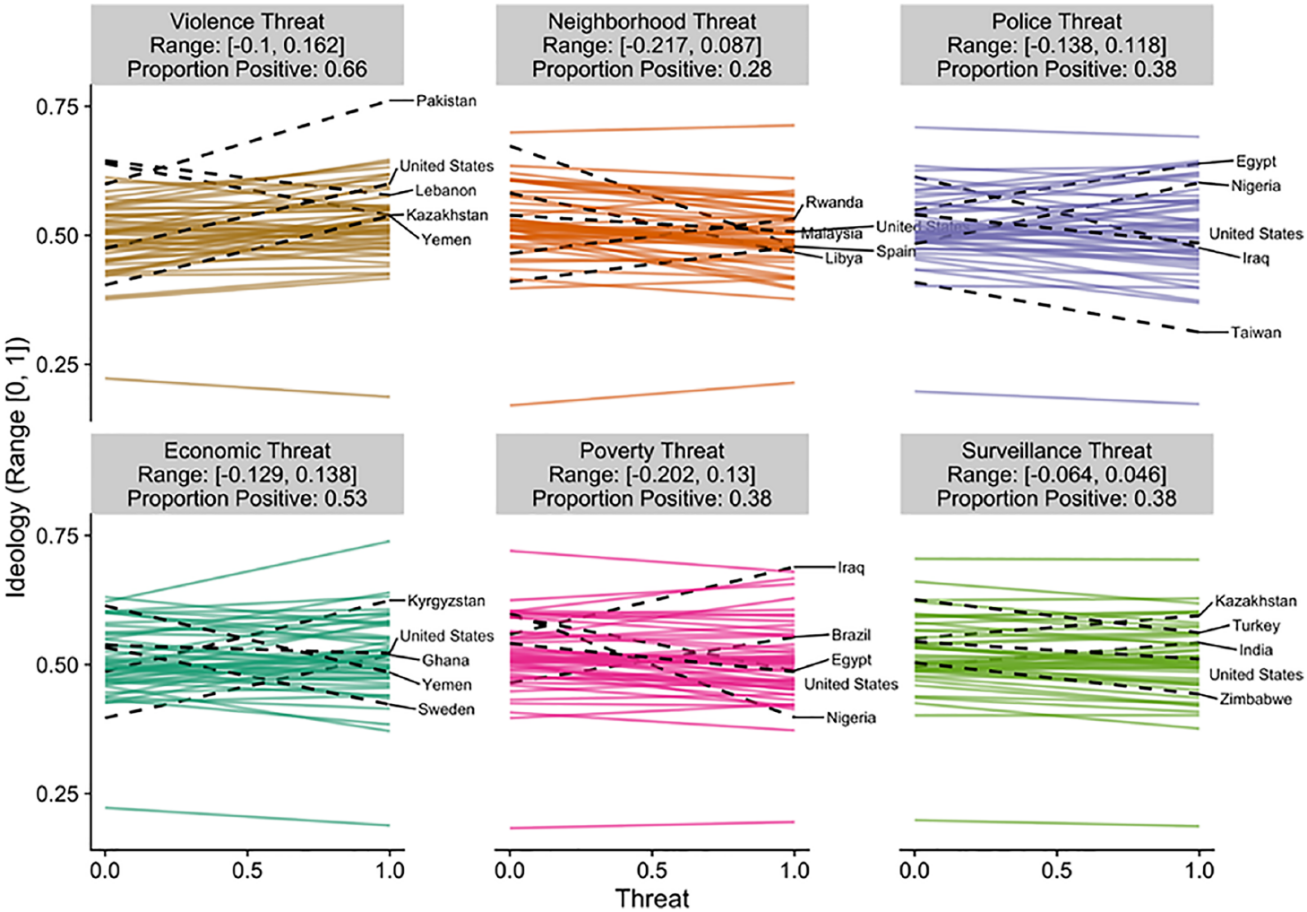
Estimated slopes between threat and ideological identification for each country. *Note.* The two largest and two smallest slopes are highlighted and labeled. The United States is also highlighted and labeled for comparison purposes. See also Table S9.

To visualize this variation, we have plotted the estimated slope for each country in Figures 2 to 6. Each figure is a different political belief. Each panel in each figure is a different threat. And each line in each panel is the estimated slope for each country. The countries with the two largest and the two smallest slopes are highlighted and labeled. The United States is also highlighted and labeled as a reference point. The title of each panel also includes the range of the slopes in the panel and the proportion of the slopes that are positive. The estimated slopes for each country can be found in Tables S5 to S9 in the Supplemental Material.

Figures 2 to 6 reveal that the findings for one country do not necessarily apply to the findings in another country. For example, Figure 6 plots the results for ideological identification. If we would have only included the United States and only focused on violence threat, we would have concluded that threat is associated with more right-wing political beliefs (*b* = .13). However, when we expand our analysis to other countries, the link between violence threat and right-wing beliefs can be quite different (range *b* = [–.10, .16]). The effect in Kazakhstan is nearly as strong as the effect in the United States, but in the opposite direction (*b* = –.10). Even maintaining our focus on the United States, by shifting our focus to other types of threats leads to different conclusions. The link between threat and right-wing ideological identification in the United States is estimated to be small and negative for every other threat we examined (range *b* = [–.05, –.02]). That is, if we would have limited our focus to violence threat and ideological identification our results would be consistent with the positive association between threat and politics assumed in the literature (e.g., Jost et al., 2003, 2017); however, if we expand beyond the United States or to different types of threats the results are inconsistent (and sometimes contrary) to this assumption. Similar comparisons can be made for each of the political beliefs we studied (e.g., by using the estimated slopes in the Supplemental Material).

### Country Characteristics

**Figure 7.**
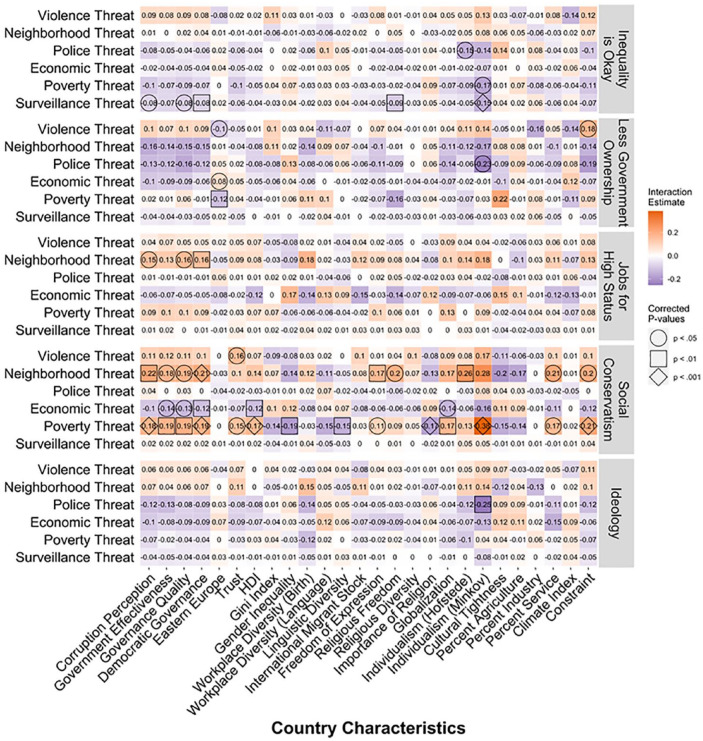
Estimates for the interaction between-ountry characteristics (*x*-axis) and threats (*y*-axis) for the five measures of political beliefs (row chunks). *Note.* Estimates that are significant using the Holm’s correction are highlighted with geometric shapes. For example, the interaction between corruption perceptions (*x*-axis) and surveillance threat (*y*-axis) when predicting ideology (bottom row chunk) is −.04 and is nonsignificant (no geometric shape). The interaction between corruption perceptions (*x*-axis) and poverty threat (*y*-axis) when predicting social conservatism (second to bottom row chunk) is .18 and is significant a *p* < .001 (diamond geometric shape).

The relationship between perceived threat and political beliefs is not consistent across countries. What explains this variation? To explore this question, we built on the multilevel models from Figure 1. We regressed each of the five political beliefs onto the six threats (country-mean centered) and their interaction with one of the country characteristics (grand-mean centered). We also controlled for region to rule out shared regional features that may be confounded with the feature of interest (Kuppens & Pollet, 2015). We included a random intercept for country and random slopes for threats. That is, in each multilevel regression we predicted the political belief with the six threats, the interaction between the six threats and the focal country characteristic, and region. The interactions are tests of whether or not the threat-politics association differs across countries. Thus, we model a cross-level interaction, using the different country characteristics (i.e., a country-level variable; see Table 1) to explain variation in the relation between threat and political beliefs at the individual-level. We do so to see the extent that country characteristic (e.g., government quality) affects the way that an individual’s political beliefs are related to their perceptions of threat. In total, we estimated 130 multilevel regression models (five political beliefs × 26 country characteristics) that tested 780 interactions (six threats × 130 regressions). The 780 interaction coefficients are in Figure 7. We adopted a Holm’s correction (Holm, 1979) for the *p* values. We applied the correction to the *p* values for each threat × political belief combination (i.e., each row in Figure 7) using the p.adjust function in R.^3^

The modal result was a nonsignificant association between the country characteristic and threat. Of the 780 interaction coefficients 45 (6%) were significant at *p* < .05, 23 (3%) were significant at *p* < .01, and eight (1%) were significant at *p* < .001 (all when applying the Holm correction for each row). The highest number of significant interactions for any country characteristic was five (Democratic Governance, Individualism [Minkov], Governance Quality). Nine country characteristics never significantly interacted with any threats (Gini index, Workplace diversity [language], International migrant stock, Religious Diversity, Cultural Tightness, Percent Agriculture, Percent Industry, Climate Index).

The country characteristics appeared to do a better job explaining variation in the association between threats and social conservatism (29 significant interactions) than in explaining variation between threats and any of the other political beliefs (16 significant interactions across the remaining political beliefs). Overall, we found few country characteristics that consistently helped explain variation in the link between threat and political beliefs. All significant interactions are plotted and can be found in the Supplemental Material.

### Digging Deeper Into Specific Country Characteristics

Table 1 highlights several country characteristics for which we had stronger expectations. Specifically, we expected that indicators of government effectiveness (Corruption Perception Index, Government Effectiveness Index, Governance Quality, and Democratic Governance Index), whether a country was a former member of the Eastern bloc, indicators of poor economic conditions (HDI, Gini Index, and Gender Inequality Index), and ideological constraint would help explain variation in the association between threat and political beliefs. We dig deeper into each of these country characteristics in the Supplemental Material and summarize those analyses here.

#### Government effectiveness

We anticipated that threat may be more strongly associated with political beliefs that give governments power, such as left-wing economic political beliefs, when a country had a well-functioning government. The only significant interaction effects between threat and country characteristics related to government effectiveness (see Table 1) when predicting economic political belief was for surveillance threat when predicting the belief that inequality is okay (see Figure 7). This negative interaction emerged for three of the government indicators; we report the simple slopes in text for the governance quality indicator. The simple slopes did not show support for the hypothesis. At high (+1 *SD*) levels of governance quality, the effect of surveillance threat was not significant (*b* = −0.01, *SE* = 0.01, *p* = .32). At low (−1 *SD*) levels of governance quality the effect was positive (*b* = 0.03, *SE* = 0.01, *p* < .001). This indicates surveillance threat are associated with a more right-wing position on the inequality is okay measure when governance quality is low, but it is unrelated to the inequality is okay measure when governance quality is high. There were no other significant interactions between threats and government effectiveness when predicting economic political beliefs. Our expectation was unsupported.

#### Eastern Bloc

We expected that threat may be more likely associated with left-wing economic political beliefs in countries from the former Eastern Bloc where state intervention in the economy was the norm. The interactions for both violence threat and poverty threat when predicting government ownership support this idea. The link between these threats and beliefs about less government ownership are negative in the former Eastern Bloc (violence threat *b* = −0.11, *SE* = 0.02, *p* < .001; poverty threat *b* = −0.15, *SE* = 0.03, *p* < .001) indicating that these threats are associated with left-wing economic beliefs in this context. The estimate for these same threats are closer to zero outside of the former Eastern Bloc (violence threat *b* = −0.008, *SE* = 0.01, *p* = .55; poverty threat *b* = −0.03, *SE* = 0.01, *p* = .03). There was also a positive interaction for economic threat when predicting government ownership that is opposite to the prediction; economic threat is positively associated with government ownership in the former Eastern Bloc (*b* = 0.07, *SE* = 0.02, *p* = .001) and near zero outside of it (*b* = −0.02, *SE* = 0.01, *p* = .10). There were no other significant interactions between threat and being a member of the Eastern Bloc. Our expectation was largely unsupported.

#### Threatening economic conditions

We anticipated that threat may be less strongly associated with right-wing political beliefs when economic conditions are poor. There was little evidence for such effects. There were no interactions with the Gini Index. There was a negative interaction between economic threat and the HDI when predicting social conservatism, opposite predictions. It showed that at high (+1 *SD*) levels of HDI the effect of economic threat was associated with left-wing views (*b* = −0.06, *SE* = 0.01, *p* < .001), but at low (−1 *SD*) levels of HDI the effect of economic threat was near zero (*b* = 0.008, *SE* = 0.01, *p* = .52). Consistent with expectations, there was a positive interaction between the HDI and poverty threat when predicting social conservatism. This suggests that poverty threat is associated with right-wing views on social conservatism (*b* = 0.06, *SE* = 0.01, *p* < .001) in countries with higher scores on the HDI; however, in countries with lower scores poverty threat is weakly associated with left-wing views on social conservatism (*b* = −0.03, *SE* = 0.01, *p* = .03). The interaction between the Gender Inequality Index and poverty threat when predicting social conservatism is also consistent with predictions. At high (+1 *SD*) levels of gender inequality the effect of poverty threat on social conservatism is near zero (*b* = −0.03, *SE* = 0.01, *p* = .08), but at low (–1SD) levels of gender inequality poverty threat is associated with more right-wing views (*b* = 0.06, *SE* = 0.01, *p* < .001). However, these two interactions were the only indicators of support for our expectation; the primary result for all combinations of threat and political beliefs was no significant interaction. Our expectation was largely unsupported.

#### Ideological constraint

We anticipated that threat may be more likely to be associated with right-wing economic political beliefs in countries with more ideological constraint. For economic political beliefs, there was one positive interaction with violence threat when predicting less government ownership, however, it was not supportive of the predictions. The effect of violence threat was unrelated to less government ownership at high (+1 *SD*) constraint (*b* = 0.01, *SE* = .02, *p* = .44). At low (−1 *SD*) constraint, violence threat was associated with left-wing views (*b* = −0.07, *SE* = .02, *p* < .001). All other possible interactions were not significant or were for cultural political beliefs. Our expectation was unsupported.

## Discussion

Threat is often associated with right-wing political beliefs in political and social psychological theory (Adorno et al., 1950; Conway et al., 2020; Jost et al., 2003). Here we sought to explore how this direct association may be more nuanced after taking into account variation in different types of political beliefs, different types of threat, and different countries. Overall, we found that the link between threat and political beliefs varied across types of political beliefs, different types of threat, and different countries. This suggests that theories that expect right-wing political beliefs to be primarily, or even typically, associated with threat are incomplete. To fully account for variation in this relationship, we need to take types of political beliefs, types of threat, and countries into account. Only taking one of these factors into account cannot account for the variation in the relationship between threat and political beliefs.

Although we identified differences between countries, we did not find much evidence for what accounts for these differences. We tested a large set of 26 country characteristics that we thought might play a role, including characteristics based on past research and a variety of other more exploratory variables. The most consistent result was that we were not able to explain much of the variation in the association between threat and political beliefs between countries. For example, there was some evidence that threat is less likely to be associated with right-wing beliefs in countries with poorer economic condition (Sibley et al., 2012), but there was also evidence directly in contrast to these findings and many null results that *cannot* be interpreted strongly. Similarly inconsistent results were found when we tested if quality government, ideological constraint, and being a part of the former Eastern Bloc accounted for variation across countries. Although there were some significant effects, overall, these factors did not play much of a role and produced results both consistent and inconsistent with expectations in Table 1. One possibility is that more specific historical circumstances or country-specific elite’s rhetorical links between threat and political beliefs may help explain the variation in the links between threat and political beliefs across countries.

The overall associations between economic and violence-related threats and political beliefs were generally (but not perfectly) consistent with expectations. Threats of violence were associated with cultural right-wing beliefs and economic threats were associated with left-wing economic political beliefs. This is consistent with prior findings that threat tends to inspire political beliefs that are perceived to help address the threat (e.g., Duckitt & Sibley, 2009; Eadeh & Chang, 2019) and turn to their ingroup when faced with threat (e.g., Voci, 2006). We had fewer expectations for surveillance and police threats because these types of threat were not considered by past literature. Here we found that the surveillance threat was associated with right-wing beliefs about government ownership. We found that police threat was associated with more left-wing cultural political beliefs, but was unrelated to economic political beliefs. Of course, as indicated in Figures 2 to 6, all of these effects were not consistent across countries.

### Implications and Future Directions

The finding that the link between threat and political beliefs depends on the type of threat, type of belief, and the country suggests that models that posit a clear link between feelings of threat and right-wing political beliefs (e.g., Hibbing et al., 2014; Jost et al., 2003) are incomplete. It is necessary to understand at least three sources of variation to understand how threat is associated with politics. Although prominent approaches, like the motivated social cognition approach to threat and ideology (Jost et al., 2003), in practice focus on a direct link between threat and politics, a broader theoretical interpretation of this work suggests a way forward. Specifically, these approaches suggest that people adopt political beliefs that fulfill their psychological needs (such as to address feelings of threat). *This general insight may still hold*. The aspect that likely does not hold is that right-wing beliefs are best suited to address all types of threat and do so to an equal degree in all countries. This means that future work aimed at developing a theory of the links between threat and political beliefs needs to identify if and how the same belief (e.g., that income inequality is okay) addresses diverse threats differently across different countries. A primary implication of our work is that this is a necessary task for all scholars with such theoretical ambitions. One way forward, may be with affordance-based models of the threat-politics association (e.g., Eadeh & Chang, 2019). These models posit that people adopt political beliefs that are best perceived as fixing a particular threat with the explicit acknowledgment that this will differ across types of threat, types of beliefs, and countries.

A theory of threat and politics is not the only direction scholars can take. Another fruitful direction is to zoom in on specific threats in specific contexts with the aim of explaining these more local relationships (e.g., the links between poverty threat and politics in the United Kingdom). Although this approach may not uncover a universal explanation for the link between threat and politics, it will likely provide a variety of insightful information. In particular, scholars should focus on threats that are not typically studied and integrate this work into political psychological approaches. For example, both surveillance and police threats are understudied, but both threats are highly relevant (see, e.g., Kodapanakkal et al., 2020). There is an increasing amount of surveillance in daily life and protests against such surveillance (e.g., in Hong Kong; Yu, 2019), moreover, police brutality has received intense scrutiny in some countries (e.g., the United States; Burch et al., 2020). Future studies should investigate diverse types of threat, including threats of government surveillance and police abuse.

Another possible direction that could also benefit from a more narrow approach, is to integrate work on threat and politics with the work on threat and challenge responses. Although not typically discussed in political psychology, this work suggests that some people respond differently to the same threat depending on the amount of resources that they have (e.g., Skinner & Brewer, 2002; Tomaka et al., 1993). If people have the resources to cope with the threat, it may be seen as more of a challenge that leads people to adopt a proactive and open-minded approach to addressing the threat. If people do not have sufficient resources, people may see the threat as more of a prototypical threat and become more risk-averse and closed-minded when addressing the threat. Although this might suggest that threats leads people with sufficient resources to become more left-wing, it is not clear that this is the correct conceptual and theoretical mapping between the two research areas. For example, when experiencing a poverty threat without sufficient resources it is not clear that taking right-wing positions on economic issues (which may increase risk in this domain) would be an effective coping mechanism. An integration of these literatures could be fruitful.

More practically, our findings hint that it is not necessarily the case that right-wing parties and other right-wing organizations will always benefit from perceived threat. Threat may be able to also push people to the left. For the enterprising politician who wants to take advantage of such findings, we think our results suggest that it is more important to be seen as solving a threat than as holding a particular type of belief. Of course, our work is just a start and not able to address this issue in full. Future research on how politicians leverage different types of threat to extract support for different types of policies could be a natural extension of our work into the field.

### Limitations

All of our analyses are based on cross-sectional data. Although we expect the relationships between threat and politics to be bi-directional (e.g., Jost et al., 2003), we cannot confirm nor disconfirm the causal direction between threat and political beliefs. Results should be interpreted as such. This is a limitation of relying on available data without the necessary longitudinal or experimental components that help to establish causality (a limitation shared with many of the studies used in meta-analyses on the topic; Jost et al., 2003, 2017).

Another limitation is that we could not choose our own measures. This meant relying on some measures with lower reliability, which may have attenuated the estimated relations. Additional indicators of threat or political beliefs may also reveal additional sources of variation. We were able to adopt measures of political beliefs used in prior studies (e.g., Malka et al., 2014, 2017), which helps our data maintain comparability with other data in this domain. One obvious shortcoming is that one measure of cultural political beliefs (jobs for high status) clearly has some overlap with economic political beliefs, making it a bit of a mix between cultural and economic political beliefs. That said, economic threats were unrelated to these beliefs suggesting that the overlap with economic was not an obvious confounding factor. Moreover, the measure of social conservatism does not have the same shortcoming. We were also able to measure more types of threat than are typical in the literature. Unfortunately, these threats may not be specifically linked with political beliefs as some perspectives might require for a strict test (e.g., Eadeh & Chang, 2019). At the same time, some of our threat measures might be seen as too overlapping with the political belief measures (e.g., poverty threat and poverty-relevant policy), leading us to overestimate the link between these threats and policies. However, if this were the case, we would not expect such extreme variation across contexts because the overlap would be similar in each place. Moreover, the ideology identification measure has no such issues.

Nonetheless, we think the data will still be informative to multiple theories of threat and politics. In particular, the key benefit of our data source is that we could focus on more countries than are typically studied when assessing the association between multiple types of threat and multiple types of political beliefs. This helps us identify variation in the link between threat and politics across countries, consistent with recent calls to diversify our samples and stimuli in political psychology (Brandt & Wagemans, 2017). Nonetheless, the number of countries was still small from the perspective of statistical power (max *N* = 56), which translated into low statistical power for identifying effects of country characteristics. Finally, like any study on politics, the results may also be affected by specific historical circumstances. This additional source of variation may further help explain the link between diverse types of threat and diverse political beliefs.

## Conclusion

Political beliefs and perceptions of threat are linked, but the relationship is nuanced. We found that the threat-politics link depends on at least three sources of variation: the type of political belief, the type of threat, and the political system. We also explored if a broad set of country characteristics could account for variation in the threat-politics link across countries. This analysis revealed few consistent results. Across all of the results, the data appear most consistent with affordance-based approaches to threat and politics (Eadeh & Chang, 2019), which suggests that people adopt political beliefs that best help address their feelings of threat.

## Supplemental Material

Brandt_Online_Appendix – Supplemental material for The Association Between Threat and Politics Depends on the Type of Threat, the Political Domain, and the Country Click here for additional data file. Supplemental material, Brandt_Online_Appendix for The Association Between Threat and Politics Depends on the Type of Threat, the Political Domain, and the Country by Mark J. Brandt, Felicity M. Turner-Zwinkels, Beste Karapirinler, Florian Van Leeuwen, Michael Bender, Yvette van Osch and Byron Adams in Personality and Social Psychology Bulletin

threatpolitics.sup.2020-04-01 – Supplemental material for The Association Between Threat and Politics Depends on the Type of Threat, the Political Domain, and the Country Click here for additional data file. Supplemental material, threatpolitics.sup.2020-04-01 for The Association Between Threat and Politics Depends on the Type of Threat, the Political Domain, and the Country by Mark J. Brandt, Felicity M. Turner-Zwinkels, Beste Karapirinler, Florian Van Leeuwen, Michael Bender, Yvette van Osch and Byron Adams in Personality and Social Psychology Bulletin

## Notes

1. We believe that there is a high likelihood of a reciprocal relationship between threat and political beliefs (e.g., Choma & Hodson, 2017; Onraet et al., 2014); however, we discuss threat as a cause of political beliefs because this is the primary way that theories in this area have conceived of the relationship. There is evidence for this causal direction (Onraet et al., 2014; Thórisdóttir & Jost, 2011).

2. Details on these measures are available in the “Methods” section.

3. The same figure, but using uncorrected *p* values is Figure S1. If the Holm’s correction is applied to all of the estimates in Figure 7, no finding is significant.
