# Supplementary material for: The Association Between Threat and Politics Depends on the Type of Threat, the Political Domain, and the Country
Source: Pers Soc Psychol Bull. 2020 Aug 26;47(2):324–43. doi: 10.1177/0146167220946187 (PMC7859575; doi:10.1177/0146167220946187)
Supplement: Brandt_Online_Appendix – Supplemental material for The Association Between Threat and Politics Depends on the Type of Threat, the Political Domain, and the Country [file Brandt_Online_Appendix.docx]

**Methodology File**

This manuscript used data from the 6ht Wave of the World Values Survey. All materials can be found at [http://worldvaluessurvey.org](http://www.worldvaluessurvey.org/)

Materials needed to replicate the results in this manuscript are available here: <https://osf.io/8zafp/?view_only=c6b3e7e56f3a4d2392a5aa5448eb26e0>
