# Supplementary material for: The Association Between Threat and Politics Depends on the Type of Threat, the Political Domain, and the Country
Source: Pers Soc Psychol Bull. 2020 Aug 26;47(2):324–43. doi: 10.1177/0146167220946187 (PMC7859575; doi:10.1177/0146167220946187)
Supplement: threatpolitics.sup.2020-04-01 – Supplemental material for The Association Between Threat and Politics Depends on the Type of Threat, the Political Domain, and the Country [file threatpolitics.sup.2020-04-01.docx]

# Supplemental Materials

Table S1

*Countries and sample sizes used in the study*

| **Country** | **N** |  | **Country** | **N** |
| --- | --- | --- | --- | --- |
| Algeria | 831 |  | Romania | 1027 |
| Argentina | 850 |  | Russia | 1101 |
| Armenia | 829 |  | Rwanda | 967 |
| Australia | 1363 |  | Singapore | 1602 |
| Azerbaijan | 882 |  | Slovenia | 842 |
| Belarus | 655 |  | South Korea | 773 |
| Brazil | 1238 |  | South Africa | 2803 |
| Cyprus (G) | 597 |  | Spain | 1006 |
| Chile | 698 |  | Sweden | 786 |
| China | 1487 |  | Taiwan | 1100 |
| Ecuador | 1195 |  | Thailand | 761 |
| Egypt | 832 |  | Trinidad and Tobago | 818 |
| Estonia | 1054 |  | Tunisia | 1030 |
| Georgia | 1038 |  | Turkey | 481 |
| Germany | 1438 |  | Ukraine | 667 |
| Ghana | 1287 |  | United States | 2079 |
| Haiti | 1677 |  | Uruguay | 751 |
| Hong Kong | 701 |  | Yemen | 724 |
| Iraq | 947 |  | India | 2369 |
| Japan | 1440 |  | Zimbabwe | 1150 |
| Jordan | 1047 |  |  |  |
| Kazakhstan | 699 |  |  |  |
| Kyrgyzstan | 583 |  |  |  |
| Lebanon | 940 |  |  |  |
| Libya | 1774 |  |  |  |
| Malaysia | 1225 |  |  |  |
| Mexico | 1960 |  |  |  |
| Morocco | 877 |  |  |  |
| Netherlands | 825 |  |  |  |
| New Zealand | 567 |  |  |  |
| Nigeria | 1393 |  |  |  |
| Pakistan | 1026 |  |  |  |
| Palestine | 833 |  |  |  |
| Peru | 954 |  |  |  |
| Philippines | 1092 |  |  |  |
| Poland | 707 |  |  |  |

# Factor Analysis of Threat Items

To reduce the number of threat items, we used exploratory principle axis factor analysis with oblimin rotation using all of the available data. The items were all country-mean centered prior to analysis. The correlation matrix for this analysis is in Table S2. A prior iteration of this manuscript only included the six “worry” items. In this version, a factor analysis indicated that the worry items formed two scales, a violence threat scale and an economic scale. The surveillance item was largely its own factor. However, based on the reviews we determined that it was worthwhile to use additional items tapping into threat that were available in the World Values Survey. When we conducted an exploratory factor analysis on the full set of items, we found four factors, but they made less theoretical sense (e.g., surveillance loaded onto the violence factor, worries about crime didn’t load onto the same factor as worries about robberies). The factors appeared to be influenced by a methods factor (i.e. the factors tended to correspond with the way the questions were asked and the location of the questions in the surveys). We also conducted an exploratory factor analysis with only the items that referred to actual threats (e.g., crime occurring in the last months, as opposed to the perception of a future threat). This also did not reveal a clean and theoretical solution.

We examined the results of the exploratory factor analyses and how the items might be expected to cohere theoretically. Based on this, we created the scales that we present in the paper. To confirm that this division of the items into scales fit the data, we specified the scales in a confirmatory factor analysis using all of the available data in lavaan (Rosseel, 2012). This indicated that the model fit the data very well (CFI = .96, TLI = .94, RMSEA = .056, 95% CI [.055, .056], SRMR = .04). All items loaded well on their predicted factor (standardized loadings > .44). Figure S1 summarizes the output of this model (figure created with lavaanPlot; Lishinski, 2018). Because this division made theoretical sense and fit the data well, we used it in our analysis.

**References**

Lishinski, A. (2018). lavaanPlot: Path Diagrams for Lavaan Models via DiagrammeR. R package version 0.5.1.https://CRAN.R-project.org/package=lavaanPlot

Rosseel, Y. (2012). lavaan: An R Package for Structural Equation Modeling. *Journal of Statistical Software, 48*, 1-36.

Table S2

*Correlation matrix used in the factor analysis of threat items.*

|  | Robbery | Police | Racism | Food | Crime | Medicine | Cash | Neighborhood. Security | Job | Education | War | Terrorism | Civil War | Surveillance |
| --- | --- | --- | --- | --- | --- | --- | --- | --- | --- | --- | --- | --- | --- | --- |
| Robbery | 1 | 0.377087 | 0.361585 | 0.162831 | 0.296324 | 0.15701 | 0.136277 | 0.356995 | 0.079763 | 0.060738 | 0.041811 | 0.039241 | 0.039134 | 0.058946 |
| Police | 0.377087 | 1 | 0.475214 | 0.159357 | 0.205924 | 0.148104 | 0.131103 | 0.192398 | 0.080135 | 0.070093 | 0.04341 | 0.047811 | 0.054396 | 0.0964 |
| Racism | 0.361585 | 0.475214 | 1 | 0.162354 | 0.207411 | 0.168062 | 0.134106 | 0.190011 | 0.083231 | 0.05586 | 0.049376 | 0.045426 | 0.049231 | 0.087109 |
| Food | 0.162831 | 0.159357 | 0.162354 | 1 | 0.41146 | 0.550678 | 0.53686 | 0.1058 | 0.082499 | 0.0721 | 0.036615 | 0.020381 | 0.033426 | 0.054615 |
| Crime | 0.296324 | 0.205924 | 0.207411 | 0.41146 | 1 | 0.421433 | 0.355707 | 0.275999 | 0.087938 | 0.087899 | 0.06329 | 0.060518 | 0.065753 | 0.081178 |
| Medicine | 0.15701 | 0.148104 | 0.168062 | 0.550678 | 0.421433 | 1 | 0.563922 | 0.125236 | 0.084264 | 0.080182 | 0.054286 | 0.046641 | 0.052379 | 0.061166 |
| Cash | 0.136277 | 0.131103 | 0.134106 | 0.53686 | 0.355707 | 0.563922 | 1 | 0.113535 | 0.125544 | 0.108258 | 0.072283 | 0.066733 | 0.069667 | 0.064109 |
| Neighborhood. Security | 0.356995 | 0.192398 | 0.190011 | 0.1058 | 0.275999 | 0.125236 | 0.113535 | 1 | 0.059471 | 0.048239 | 0.03112 | 0.040394 | 0.042374 | 0.038265 |
| Job | 0.079763 | 0.080135 | 0.083231 | 0.082499 | 0.087938 | 0.084264 | 0.125544 | 0.059471 | 1 | 0.546729 | 0.359832 | 0.33242 | 0.338188 | 0.31447 |
| Education | 0.060738 | 0.070093 | 0.05586 | 0.0721 | 0.087899 | 0.080182 | 0.108258 | 0.048239 | 0.546729 | 1 | 0.435953 | 0.40189 | 0.400726 | 0.322986 |
| War | 0.041811 | 0.04341 | 0.049376 | 0.036615 | 0.06329 | 0.054286 | 0.072283 | 0.03112 | 0.359832 | 0.435953 | 1 | 0.761329 | 0.728333 | 0.439453 |
| Terrorism | 0.039241 | 0.047811 | 0.045426 | 0.020381 | 0.060518 | 0.046641 | 0.066733 | 0.040394 | 0.33242 | 0.40189 | 0.761329 | 1 | 0.772662 | 0.458862 |
| Civil War | 0.039134 | 0.054396 | 0.049231 | 0.033426 | 0.065753 | 0.052379 | 0.069667 | 0.042374 | 0.338188 | 0.400726 | 0.728333 | 0.772662 | 1 | 0.487256 |
| Surveillance | 0.058946 | 0.0964 | 0.087109 | 0.054615 | 0.081178 | 0.061166 | 0.064109 | 0.038265 | 0.31447 | 0.322986 | 0.439453 | 0.458862 | 0.487256 | 1 |

*
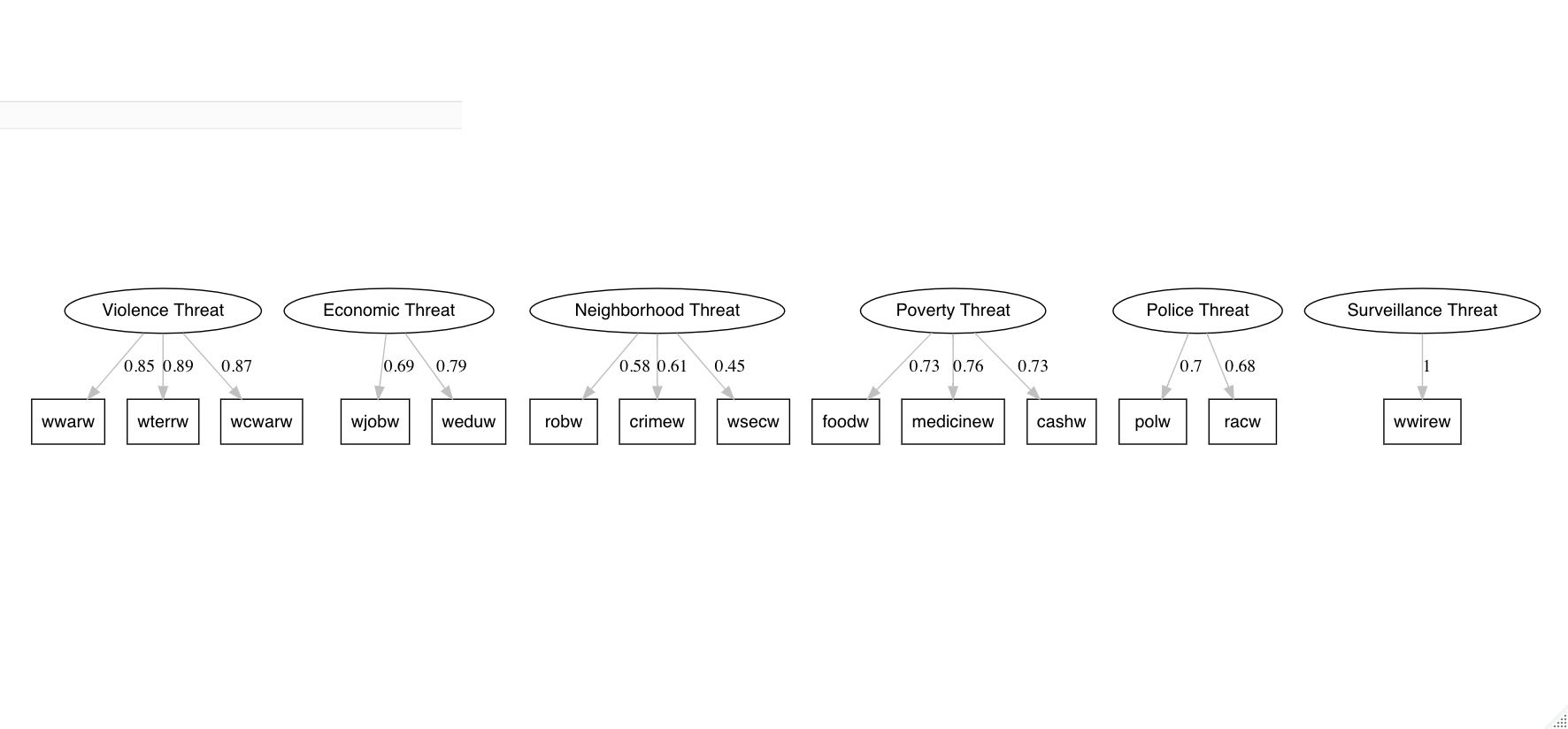
*

**Figure S1. Summary of CFA of threat items**

*Note*: Loadings are standardized. All latent variables were also allowed to covary, but the associations are not presented to maintain clarity.

# Factor Analysis of Political Belief Items

We used a principle axis exploratory factor analysis and oblimin rotation to assess the structure of the political belief items (country-mean centered; correlation matrix in Table S3). A parallel analysis identified three factors, whereby both ideology and government ownership did not clearly load another any one factor (all loadings for these two items were < .3). A confirmatory factor analysis indicated that this model fit the data well (CFI = .99, TLI = .97, RMSEA = .020, 95%CI [.018, .022], SRMR = .011). The model is summarized in Figure S2.

*Table S3*

*Correlation matrix used in the factor analysis of for political beliefs items*

|  | Ideology | Gay | Abortion | Men Jobs | Immigrants Jobs | Income inequality | Gov. Ownership | Gov. Responsibility |
| --- | --- | --- | --- | --- | --- | --- | --- | --- |
| Ideology | 1 | 0.05235 | 0.055756 | 0.058773 | 0.039074 | 0.138825 | -0.00559 | 0.12016 |
| Gay | 0.05235 | 1 | 0.477371 | 0.098656 | 0.083434 | 0.01024 | -0.00834 | -0.02981 |
| Abortion | 0.055756 | 0.477371 | 1 | 0.051926 | 0.048203 | 0.019518 | -0.00033 | -0.01741 |
| Men Jobs | 0.058773 | 0.098656 | 0.051926 | 1 | 0.189444 | 0.004328 | -0.01221 | -0.01278 |
| Immigrants Jobs | 0.039074 | 0.083434 | 0.048203 | 0.189444 | 1 | 0.014217 | -0.01572 | -0.03337 |
| Income inequality | 0.138825 | 0.01024 | 0.019518 | 0.004328 | 0.014217 | 1 | -0.07548 | 0.227841 |
| Gov. Ownership | -0.00559 | -0.00834 | -0.00033 | -0.01221 | -0.01572 | -0.07548 | 1 | -0.02033 |
| Gov. Responsibility | 0.12016 | -0.02981 | -0.01741 | -0.01278 | -0.03337 | 0.227841 | -0.02033 | 1 |


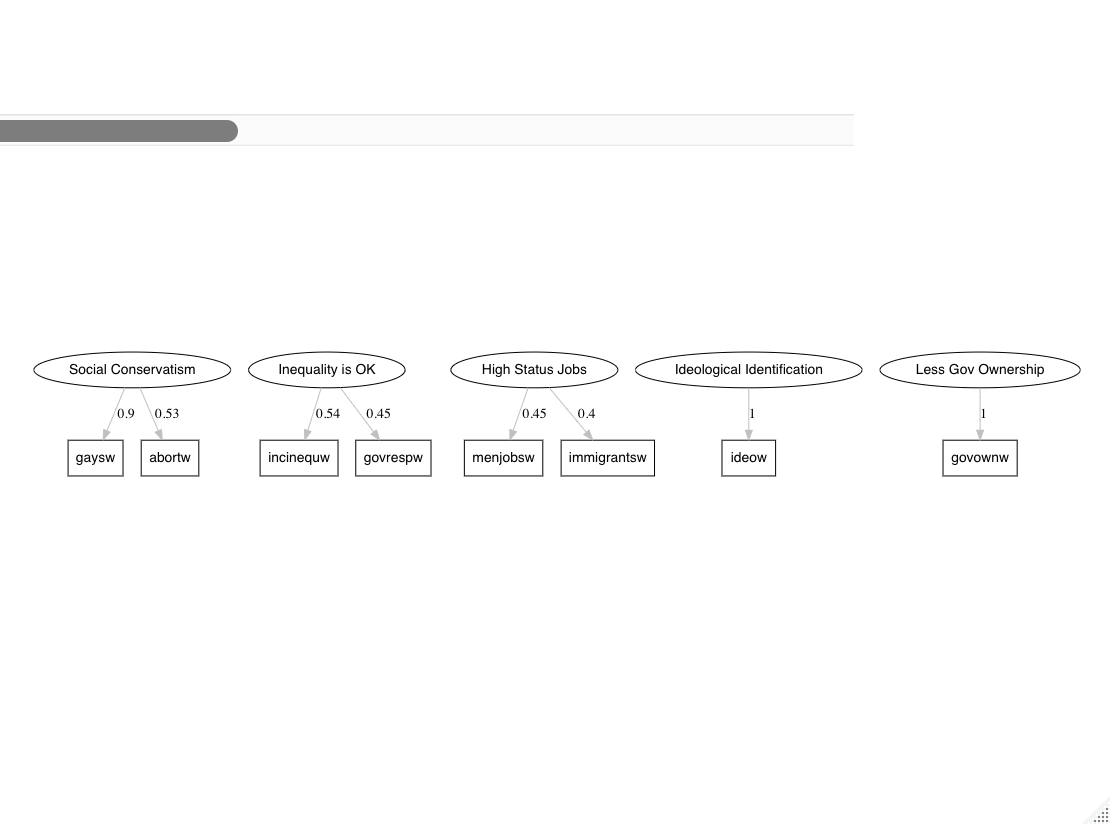


**Figure S2. Summary of CFA of political items**

*Note*: Loadings are standardized. All latent variables were also allowed to covary, but the associations are not presented to maintain clarity.

Table S4

*Correlation matrix of the country-level variables*

|  | 1 | 2 | 3 | 4 | 5 | 6 | 7 | 8 | 9 | 10 | 11 | 12 | 13 | 14 | 15 | 16 | 17 | 18 | 19 | 20 | 21 | 22 | 23 | 24 | 25 | 26 |
| --- | --- | --- | --- | --- | --- | --- | --- | --- | --- | --- | --- | --- | --- | --- | --- | --- | --- | --- | --- | --- | --- | --- | --- | --- | --- | --- |
| Gini Index |  |  |  |  |  |  |  |  |  |  |  |  |  |  |  |  |  |  |  |  |  |  |  |  |  |  |
| HDI | -0.29 |  |  |  |  |  |  |  |  |  |  |  |  |  |  |  |  |  |  |  |  |  |  |  |  |  |
| Religious Freedom | 0.18 | 0.35 |  |  |  |  |  |  |  |  |  |  |  |  |  |  |  |  |  |  |  |  |  |  |  |  |
| Gender Inequality | 0.39 | -0.88 | -0.35 |  |  |  |  |  |  |  |  |  |  |  |  |  |  |  |  |  |  |  |  |  |  |  |
| Governance Quality | -0.27 | 0.77 | 0.42 | -0.71 |  |  |  |  |  |  |  |  |  |  |  |  |  |  |  |  |  |  |  |  |  |  |
| Cultural Tightness | 0.12 | -0.50 | -0.66 | 0.43 | -0.12 |  |  |  |  |  |  |  |  |  |  |  |  |  |  |  |  |  |  |  |  |  |
| Democratic Governance | -0.18 | 0.74 | 0.59 | -0.62 | 0.89 | -0.46 |  |  |  |  |  |  |  |  |  |  |  |  |  |  |  |  |  |  |  |  |
| Corruption Perception | -0.15 | 0.72 | 0.45 | -0.64 | 0.88 | -0.35 | 0.88 |  |  |  |  |  |  |  |  |  |  |  |  |  |  |  |  |  |  |  |
| Individualism (Minkov) | -0.28 | 0.87 | 0.45 | -0.84 | 0.77 | -0.57 | 0.80 | 0.71 |  |  |  |  |  |  |  |  |  |  |  |  |  |  |  |  |  |  |
| Individualism (Hofstede) | -0.41 | 0.52 | 0.35 | -0.45 | 0.69 | -0.68 | 0.73 | 0.64 | 0.72 |  |  |  |  |  |  |  |  |  |  |  |  |  |  |  |  |  |
| Globalization | -0.26 | 0.83 | 0.32 | -0.75 | 0.86 | -0.40 | 0.81 | 0.82 | 0.78 | 0.59 |  |  |  |  |  |  |  |  |  |  |  |  |  |  |  |  |
| International Migrant Stock | -0.33 | 0.49 | 0.24 | -0.44 | 0.44 | -0.17 | 0.30 | 0.45 | 0.35 | 0.31 | 0.44 |  |  |  |  |  |  |  |  |  |  |  |  |  |  |  |
| Climate Index | -0.48 | 0.27 | -0.21 | -0.36 | 0.01 | -0.29 | -0.06 | -0.04 | 0.26 | 0.25 | 0.15 | 0.08 |  |  |  |  |  |  |  |  |  |  |  |  |  |  |
| Freedom of Expression | 0.07 | 0.56 | 0.61 | -0.38 | 0.61 | -0.70 | 0.81 | 0.66 | 0.48 | 0.46 | 0.64 | 0.09 | -0.28 |  |  |  |  |  |  |  |  |  |  |  |  |  |
| Religious Diversity | -0.43 | 0.44 | 0.48 | -0.42 | 0.47 | -0.14 | 0.51 | 0.53 | 0.24 | 0.27 | 0.43 | 0.51 | 0.08 | 0.34 |  |  |  |  |  |  |  |  |  |  |  |  |
| Importance of Religion | 0.39 | -0.69 | -0.41 | 0.73 | -0.49 | 0.62 | -0.55 | -0.52 | -0.76 | -0.48 | -0.54 | -0.48 | -0.47 | -0.29 | -0.54 |  |  |  |  |  |  |  |  |  |  |  |
| Workplace Diversity (Language) | -0.24 | -0.46 | -0.39 | 0.26 | -0.21 | 0.46 | -0.47 | -0.21 | -0.40 | -0.29 | -0.22 | 0.19 | -0.17 | -0.48 | -0.25 | 0.40 |  |  |  |  |  |  |  |  |  |  |
| Workplace Diversity (Birth) | -0.23 | 0.48 | 0.35 | -0.37 | 0.51 | -0.10 | 0.36 | 0.59 | 0.24 | 0.39 | 0.46 | 0.92 | -0.25 | 0.22 | 0.45 | -0.24 | 0.26 |  |  |  |  |  |  |  |  |  |
| Linguistic Diversity | 0.09 | -0.37 | -0.01 | 0.31 | -0.22 | 0.49 | -0.25 | -0.15 | -0.60 | -0.31 | -0.23 | -0.08 | -0.20 | -0.16 | 0.22 | 0.36 | 0.80 | 0.10 |  |  |  |  |  |  |  |  |
| Government Effectiveness | -0.15 | 0.80 | 0.45 | -0.72 | 0.90 | -0.21 | 0.88 | 0.92 | 0.70 | 0.50 | 0.83 | 0.47 | -0.04 | 0.66 | 0.59 | -0.52 | -0.21 | 0.58 | -0.14 |  |  |  |  |  |  |  |
| Percent Agriculture | 0.15 | -0.80 | -0.23 | 0.60 | -0.60 | 0.54 | -0.60 | -0.51 | -0.68 | -0.42 | -0.67 | -0.47 | -0.18 | -0.51 | -0.43 | 0.56 | 0.52 | -0.46 | 0.22 | -0.59 |  |  |  |  |  |  |
| Percent Industry | -0.34 | 0.40 | -0.22 | -0.43 | 0.19 | 0.14 | 0.07 | 0.07 | 0.19 | -0.06 | 0.35 | 0.00 | 0.42 | 0.06 | 0.10 | -0.23 | -0.27 | -0.26 | -0.20 | 0.11 | -0.54 |  |  |  |  |  |
| Percent Service | -0.05 | 0.77 | 0.37 | -0.54 | 0.61 | -0.50 | 0.66 | 0.58 | 0.58 | 0.42 | 0.64 | 0.55 | 0.04 | 0.58 | 0.48 | -0.57 | -0.37 | 0.53 | -0.19 | 0.65 | -0.94 | 0.21 |  |  |  |  |
| Eastern Europe | -0.64 | 0.20 | -0.10 | -0.35 | -0.25 | -0.40 | -0.22 | -0.21 | -0.02 | 0.11 | 0.11 | 0.01 | 0.81 | -0.24 | -0.24 | -0.38 | -0.25 | -0.31 | -0.18 | -0.24 | 0.11 | 0.29 | -0.26 |  |  |  |
| Trust | -0.26 | 0.43 | 0.20 | -0.46 | 0.48 | -0.35 | 0.42 | 0.44 | 0.64 | 0.54 | 0.33 | 0.38 | 0.36 | 0.05 | 0.48 | -0.68 | -0.02 | 0.27 | -0.17 | 0.44 | -0.36 | 0.08 | 0.40 | -0.03 |  |  |
| Constrain | -0.11 | 0.50 | 0.28 | -0.43 | 0.65 | -0.27 | 0.70 | 0.62 | 0.69 | 0.60 | 0.53 | 0.16 | 0.05 | 0.46 | 0.19 | -0.37 | -0.27 | 0.27 | -0.24 | 0.53 | -0.42 | 0.03 | 0.48 | -0.38 | 0.43 |  |

Table S5

Estimated intercepts and slopes for inequality is okay.

| country | Intercept | Surveillance Threat | Violence Threat | Economic Threat | Poverty Threat | Neighborhood Threat | Police Threat |
| --- | --- | --- | --- | --- | --- | --- | --- |
| Algeria | 0.463144 | 0.036024 | 0.083955 | -0.03908 | -0.06533 | -0.06761 | 0.040694 |
| Azerbaijan | 0.500723 | -0.02135 | -0.11784 | -0.09492 | 0.04374 | 0.000239 | -0.00207 |
| Argentina | 0.426412 | 0.03766 | 0.15012 | -0.05386 | -0.06536 | -0.03344 | 0.030967 |
| Australia | 0.48001 | -0.03709 | 0.024753 | -0.05861 | -0.1417 | -0.03622 | -0.0766 |
| Armenia | 0.371286 | 0.043148 | -0.01067 | 0.032506 | -0.04195 | -0.01983 | 0.056729 |
| Brazil | 0.394679 | 0.012438 | 0.070236 | 0.011075 | -0.04115 | -0.03447 | 0.051627 |
| Belarus | 0.365639 | 0.059914 | -0.04973 | 0.016178 | -0.08443 | 0.007451 | 0.017709 |
| Chile | 0.30611 | -0.03952 | 0.060622 | -0.0015 | -0.07817 | 0.018421 | -0.00478 |
| China | 0.41081 | 0.010762 | -0.00799 | -0.01827 | -0.08441 | -0.06628 | 0.052344 |
| Taiwan | 0.514512 | -0.00883 | 0.015122 | -0.08324 | -0.06393 | -0.0367 | 0.001643 |
| Cyprus (G) | 0.268852 | 0.021809 | 0.034569 | 0.011743 | -0.03992 | -0.01744 | 0.09423 |
| Ecuador | 0.50606 | -0.02257 | 0.006405 | -0.1619 | -0.0462 | -0.05188 | -0.00202 |
| Estonia | 0.314101 | -0.01395 | 0.044173 | -0.00813 | -0.14931 | -0.06703 | -0.01976 |
| Georgia | 0.397559 | 0.011845 | 0.083641 | -0.0394 | -0.05652 | -0.02864 | -0.0091 |
| Palestine | 0.421856 | 0.000921 | 0.047519 | 0.004954 | -0.08167 | -0.03252 | 0.004959 |
| Germany | 0.356073 | -0.02615 | -0.00139 | -0.02359 | -0.04879 | 0.005157 | -0.01001 |
| Ghana | 0.543951 | -0.01171 | 0.034029 | -0.02539 | -0.0582 | 0.010488 | -0.032 |
| Haiti | 0.29145 | 0.006896 | -0.04857 | 0.01561 | -0.02852 | 0.010217 | -0.00698 |
| Hong Kong | 0.55748 | -0.01832 | 0.003515 | -0.03011 | -0.0861 | -0.021 | -0.03317 |
| India | 0.240729 | 0.069954 | -0.0143 | -0.04969 | -0.02152 | 0.023478 | 0.082212 |
| Iraq | 0.348132 | 0.042564 | 0.019008 | -0.01813 | -0.06974 | -0.01793 | -0.07485 |
| Japan | 0.394906 | -0.03172 | -0.01238 | -0.08381 | -0.06812 | 0.000146 | 0.049208 |
| Kazakhstan | 0.41875 | 0.045653 | -0.08174 | 0.001872 | -0.05692 | 0.001675 | 0.023356 |
| Jordan | 0.497565 | 0.010692 | -0.00672 | -0.01925 | -0.05099 | -0.03316 | 0.016431 |
| South Korea | 0.441878 | -0.01756 | 0.049426 | -0.09747 | -0.03716 | -0.01351 | -0.04805 |
| Kyrgyzstan | 0.475505 | -0.02593 | -0.09499 | 0.021922 | -0.00753 | 0.019184 | 0.053879 |
| Lebanon | 0.45546 | 0.007258 | -0.03748 | -0.02817 | 0.053499 | 0.003584 | 0.101306 |
| Libya | 0.47194 | -0.01528 | 0.027958 | -0.01712 | -0.08871 | -0.01163 | 0.0184 |
| Malaysia | 0.591339 | 0.060973 | 0.196652 | 0.025946 | -0.10989 | -0.09665 | 0.075542 |
| Mexico | 0.442113 | -0.02231 | -0.02469 | -0.03326 | -0.07012 | 0.002181 | 0.020443 |
| Morocco | 0.405115 | 0.02221 | 0.132148 | -0.04426 | -0.10144 | -0.0527 | -0.00805 |
| Netherlands | 0.528711 | -0.02779 | 0.106964 | -0.07091 | -0.12482 | -0.03816 | -0.04517 |
| New Zealand | 0.539762 | -0.05438 | 0.075137 | -0.0585 | -0.09956 | -0.01119 | -0.07276 |
| Nigeria | 0.494783 | 0.05613 | -0.02614 | -0.04038 | -0.04627 | -0.01501 | 0.021656 |
| Pakistan | 0.571572 | 0.029895 | 0.113321 | 0.007942 | -0.16952 | -0.03896 | 0.179703 |
| Peru | 0.490127 | 0.039123 | 0.033662 | -0.01151 | -0.05564 | -0.03166 | 0.040154 |
| Philippines | 0.57846 | -0.02806 | 0.047455 | -0.0223 | -0.00608 | -0.03753 | 0.029818 |
| Poland | 0.49416 | 0.0111 | -0.02512 | 0.02475 | -0.08346 | -0.01041 | -0.03252 |
| Romania | 0.527497 | 0.02808 | 0.068271 | 0.000972 | -0.08654 | -0.06773 | 0.035306 |
| Russia | 0.265814 | 0.065361 | -0.12653 | 0.071192 | -0.05851 | 0.004719 | 0.102737 |
| Rwanda | 0.347509 | 0.002685 | -0.03442 | -0.02284 | -0.03664 | 0.019901 | 0.011811 |
| Singapore | 0.50354 | 5.96E-06 | -0.00825 | -0.02269 | -0.08052 | -0.01488 | 0.038135 |
| Slovenia | 0.314642 | 0.028569 | -0.04506 | -0.03939 | -0.09024 | 0.021255 | 0.06349 |
| South Africa | 0.548945 | 0.053899 | 0.05147 | -0.04343 | -0.04487 | -0.09472 | 0.069609 |
| Zimbabwe | 0.488007 | -0.00504 | -0.06266 | -0.04503 | -0.08057 | 0.020419 | -0.03927 |
| Spain | 0.419603 | 0.034872 | -0.02747 | 0.013277 | -0.0146 | -0.00797 | 0.035219 |
| Sweden | 0.481368 | -0.02785 | 0.083694 | -0.11428 | -0.13093 | -0.00466 | -0.03009 |
| Thailand | 0.436486 | 0.02529 | -0.09212 | -0.04525 | 0.036542 | -0.01164 | -0.00463 |
| Trinidad and Tobago | 0.611305 | 0.033847 | -0.02368 | 0.011305 | -0.09437 | -0.04051 | 0.01138 |
| Tunisia | 0.405733 | 0.050376 | 0.026994 | -0.10343 | -0.12104 | -0.05116 | 0.003413 |
| Turkey | 0.364343 | 0.003884 | 0.039087 | -0.01041 | -0.06911 | 0.033614 | -0.01862 |
| Ukraine | 0.248285 | 0.072632 | -0.056 | 0.00709 | -0.06301 | 0.004617 | 0.107801 |
| Egypt | 0.310941 | 0.061604 | -0.10266 | 0.020084 | -0.04716 | -0.04966 | 0.035622 |
| United States | 0.551427 | -0.03169 | 0.070478 | -0.08801 | -0.1318 | -0.01169 | -0.07352 |
| Uruguay | 0.457855 | 0.014066 | 0.009572 | 0.071948 | -0.12982 | -0.04239 | 0.019586 |
| Yemen | 0.398046 | -0.02864 | 0.196569 | -0.04552 | -0.08492 | -0.05878 | -0.01042 |

Table S6

Estimated intercepts and slopes for government ownership.

| country | Intercept | Surveillance Threat | Violence Threat | Economic Threat | Poverty Threat | Neighborhood Threat | Police Threat |
| --- | --- | --- | --- | --- | --- | --- | --- |
| Algeria | 0.467419 | 0.084456 | -0.05571 | -0.06904 | 0.045365 | 0.014878 | -0.01637 |
| Azerbaijan | 0.532431 | 0.012095 | -0.0244 | 0.019607 | -0.12379 | 0.027801 | 0.016339 |
| Argentina | 0.417166 | 0.026823 | 0.111231 | -0.0296 | 0.026706 | -0.00708 | -0.07005 |
| Australia | 0.583188 | -0.0096 | 0.011986 | -0.05521 | -0.02536 | -0.02615 | -0.06578 |
| Armenia | 0.421907 | 0.052152 | -0.05684 | 0.031336 | -0.13648 | 0.010473 | 0.124345 |
| Brazil | 0.525787 | 0.024407 | -0.00796 | -0.00839 | -0.2382 | -0.05091 | 0.079858 |
| Belarus | 0.560285 | 0.12366 | -0.1965 | 0.082087 | -0.08035 | 0.13653 | 0.145716 |
| Chile | 0.344394 | -0.00134 | 0.112145 | -0.03982 | 0.072062 | -0.03839 | -0.00732 |
| China | 0.450967 | 0.046579 | -0.09374 | -0.00156 | -0.00781 | 0.176737 | 0.09425 |
| Taiwan | 0.556962 | 0.047849 | -0.0675 | -0.01924 | -0.07872 | -0.02889 | 0.056336 |
| Cyprus (G) | 0.583831 | -0.0231 | 0.010277 | -0.07324 | 9.45E-05 | -0.00434 | -0.07031 |
| Ecuador | 0.474366 | 0.058208 | -0.07242 | 0.114747 | -0.09757 | 0.032944 | 0.018598 |
| Estonia | 0.472602 | 0.028695 | -0.1001 | 0.060117 | -0.10056 | -0.08073 | 0.01052 |
| Georgia | 0.545384 | 0.037872 | -0.05619 | 0.063223 | -0.1542 | -0.00928 | 0.043142 |
| Palestine | 0.464312 | -0.03402 | 0.000325 | 0.005924 | 0.076815 | 0.049239 | -0.00679 |
| Germany | 0.526888 | -0.03871 | 0.012176 | 0.010185 | -0.03387 | -0.00823 | -0.05427 |
| Ghana | 0.485524 | 0.009133 | 0.045939 | 0.040168 | -0.13378 | -0.0191 | 0.024915 |
| Haiti | 0.6197 | 0.005898 | 0.059196 | 0.000678 | -0.06126 | -0.03048 | -0.00024 |
| Hong Kong | 0.463581 | 0.009629 | -0.03097 | 0.01683 | -0.02637 | 0.050827 | 0.019297 |
| India | 0.532911 | 0.061774 | -0.13424 | -0.02496 | -0.08008 | 0.060608 | 0.086895 |
| Iraq | 0.446142 | -0.03509 | -0.09225 | 0.063496 | -0.07363 | 0.044339 | 0.139312 |
| Japan | 0.610575 | 0.021065 | -0.03097 | 0.014853 | -0.04733 | -0.0689 | -0.02724 |
| Kazakhstan | 0.349874 | 0.000411 | -0.06436 | 0.088974 | -0.09005 | 0.066361 | 0.10781 |
| Jordan | 0.361046 | 0.038907 | -0.01861 | -0.01692 | -0.0239 | -0.00588 | 0.041533 |
| South Korea | 0.504835 | 0.013351 | -0.05613 | 0.002426 | -0.05786 | -0.00431 | -0.00158 |
| Kyrgyzstan | 0.507836 | 0.004826 | -0.10524 | 0.016371 | -0.14504 | -0.0765 | 0.012887 |
| Lebanon | 0.522113 | 0.073701 | -0.00193 | -0.03785 | -0.15919 | -0.03597 | -0.03797 |
| Libya | 0.450319 | 0.032026 | -0.03469 | -0.01619 | -0.0205 | 0.0963 | -0.07328 |
| Malaysia | 0.43348 | -0.03966 | -0.08934 | -0.11406 | 0.013486 | 0.173255 | 0.037902 |
| Mexico | 0.4388 | 0.022251 | -0.01589 | 0.02727 | -0.07138 | -0.00344 | -0.0259 |
| Morocco | 0.505398 | -0.00889 | 0.211993 | 0.001122 | 0.060154 | -0.06494 | -0.11122 |
| Netherlands | 0.50421 | -0.01526 | 0.003055 | -0.00668 | -0.09641 | -0.04397 | 0.038975 |
| New Zealand | 0.612547 | 0.020432 | 0.002873 | -0.0644 | -0.0588 | -0.05817 | -0.11227 |
| Nigeria | 0.495906 | -0.04233 | -0.05225 | 0.046038 | -0.13948 | 0.061463 | 0.118509 |
| Pakistan | 0.474964 | 0.052737 | -0.04794 | -0.04714 | 0.235524 | 0.027853 | -0.32047 |
| Peru | 0.454014 | 0.037518 | 0.002466 | -0.04038 | 0.017866 | 0.011475 | 0.040164 |
| Philippines | 0.410401 | 0.039745 | -0.04958 | -0.01238 | -0.12195 | 0.050998 | 0.013802 |
| Poland | 0.422854 | 0.051436 | -0.14449 | 0.027571 | -0.16899 | 0.059446 | 0.09211 |
| Romania | 0.455097 | 0.019319 | -0.09286 | 0.017305 | -0.1461 | 0.006404 | -0.06233 |
| Russia | 0.408329 | -0.01461 | -0.12553 | 0.07943 | -0.17066 | 0.059865 | 0.064522 |
| Rwanda | 0.667911 | -0.00275 | 0.045927 | -0.03166 | -0.00324 | -0.03611 | -0.02115 |
| Singapore | 0.545076 | 0.028112 | -0.03023 | -0.03162 | -0.03258 | -0.06132 | -0.03696 |
| Slovenia | 0.566221 | 0.021884 | -0.07141 | 0.013242 | -0.19848 | 0.022264 | 0.031588 |
| South Africa | 0.421726 | -0.00037 | -0.08577 | 0.018615 | -0.03167 | 0.124794 | -0.08678 |
| Zimbabwe | 0.58095 | 0.061769 | 0.014707 | -0.0444 | -0.08464 | 0.094297 | 0.048721 |
| Spain | 0.511167 | 0.044318 | 0.003378 | 0.011243 | -0.02765 | -0.02364 | -0.08706 |
| Sweden | 0.53038 | -0.00803 | 0.100644 | -0.00365 | -0.05353 | -0.06601 | -0.09364 |
| Thailand | 0.392778 | 0.008451 | -0.17757 | 0.032466 | -0.13558 | 0.088023 | 0.134936 |
| Trinidad and Tobago | 0.512038 | 0.009739 | -0.03603 | -0.02232 | -0.0341 | -0.02786 | 0.030241 |
| Tunisia | 0.459397 | 0.044574 | -0.04405 | -0.0322 | 0.038938 | 0.002663 | -0.0253 |
| Turkey | 0.504634 | 0.026694 | 0.100637 | -0.05575 | 0.018318 | -0.08755 | -0.06821 |
| Ukraine | 0.39365 | -0.00445 | -0.1013 | 0.118502 | -0.09159 | 0.064963 | 0.065175 |
| Egypt | 0.360594 | 0.049537 | 0.081946 | -0.05768 | 0.128893 | -0.14629 | 0.036433 |
| United States | 0.712144 | 0.002614 | 0.053574 | -0.08348 | -0.07784 | -0.09741 | -0.1008 |
| Uruguay | 0.500135 | 0.033588 | 0.045102 | -0.13822 | -0.02035 | -0.0177 | -0.11506 |
| Yemen | 0.374286 | -0.00887 | -0.18555 | 0.163237 | -0.08046 | 0.09278 | 0.303331 |

Table S7

Estimated intercepts and slopes for jobs for high status.

| country | Intercept | Surveillance Threat | Violence Threat | Economic Threat | Poverty Threat | Neighborhood Threat | Police Threat |
| --- | --- | --- | --- | --- | --- | --- | --- |
| Azerbaijan | 0.880019 | -0.04803 | 0.071513 | -0.02945 | -0.02536 | -0.02823 | -0.06646 |
| Argentina | 0.402194 | -0.01657 | 0.121968 | 0.066645 | 0.132622 | 0.049667 | -0.01357 |
| Australia | 0.382026 | 0.018717 | 0.160688 | -0.06249 | 0.070665 | 0.075446 | -0.03793 |
| Armenia | 0.72285 | 0.036504 | 0.120079 | -0.05338 | 0.079382 | -0.02911 | 0.050619 |
| Brazil | 0.497663 | -0.00679 | 0.053424 | -0.03398 | 0.036353 | -0.05586 | -0.04453 |
| Belarus | 0.568253 | -0.01664 | 0.041431 | -0.03863 | 0.050833 | 0.024973 | 0.030634 |
| Chile | 0.530882 | -0.02607 | 0.05645 | 0.016122 | 0.147197 | 0.03511 | 0.041376 |
| China | 0.623957 | 0.00304 | 0.051751 | -0.03801 | 0.046344 | -0.10218 | -0.0766 |
| Taiwan | 0.692465 | -0.00905 | 0.104496 | 0.016843 | 0.064574 | 0.021602 | -0.03985 |
| Cyprus (G) | 0.605721 | -0.01452 | 0.078583 | -0.06718 | -0.04697 | -0.0051 | -0.05438 |
| Ecuador | 0.559192 | 0.008729 | 0.024913 | -0.06798 | 0.083665 | 0.005707 | 0.019681 |
| Estonia | 0.568766 | -0.00648 | 0.035286 | -0.04286 | 0.053431 | 0.007831 | 0.061303 |
| Georgia | 0.68363 | -0.00614 | 0.088588 | -0.0065 | 0.042304 | -0.05971 | -0.06356 |
| Germany | 0.398018 | -0.01883 | 0.014688 | 0.026796 | 0.124224 | 0.059965 | 0.001595 |
| Ghana | 0.692083 | -0.00614 | -0.05422 | 0.061401 | 0.050045 | -0.01653 | -0.05559 |
| Haiti | 0.640663 | -0.01873 | 0.020785 | -0.03077 | 0.006016 | -0.03323 | -0.00779 |
| Hong Kong | 0.663979 | -0.02479 | 0.066099 | 0.013534 | 0.071778 | -0.0179 | -0.02985 |
| India | 0.74741 | -0.0542 | -0.02521 | 0.132942 | 0.089001 | 0.007398 | -0.04023 |
| Japan | 0.704815 | -0.01127 | 0.036683 | -0.01316 | 0.091419 | -0.03332 | -0.04463 |
| Kazakhstan | 0.697607 | 0.009535 | 0.030885 | -0.14844 | -0.02145 | -0.04834 | -0.10063 |
| Jordan | 0.886426 | -0.00766 | 0.007965 | -0.01252 | 0.005687 | -0.05582 | -0.01271 |
| South Korea | 0.670459 | -0.00201 | 0.044935 | 0.034701 | 0.073541 | -0.08607 | -0.06565 |
| Kyrgyzstan | 0.641733 | 0.023725 | 0.089493 | -0.0593 | 0.033259 | -0.01586 | 0.04772 |
| Libya | 0.831309 | -0.00792 | 0.003174 | 0.012419 | 0.004437 | -0.05081 | -0.08127 |
| Malaysia | 0.820299 | -0.03401 | 0.045477 | 0.015732 | -0.01768 | -0.03743 | -0.02649 |
| Mexico | 0.460421 | 0.007049 | 0.067668 | -0.04032 | 0.11963 | -0.06578 | 0.02322 |
| Morocco | 0.720707 | -0.00256 | 0.128735 | 0.164186 | 0.244584 | -0.05823 | 0.022002 |
| Netherlands | 0.322713 | -0.00077 | 0.067843 | 0.062314 | 0.13761 | 0.059264 | -0.02527 |
| New Zealand | 0.365497 | 0.005502 | 0.093377 | -0.04689 | 0.103487 | 0.059915 | 0.056278 |
| Nigeria | 0.747092 | 0.0152 | 0.090688 | 0.013627 | 0.039453 | -0.08584 | -0.03258 |
| Pakistan | 0.754813 | -0.06727 | 0.138197 | 0.264218 | -0.04032 | 0.051393 | -0.11619 |
| Peru | 0.533409 | -0.03287 | -0.0155 | 0.05032 | 0.037604 | 0.005076 | -0.02935 |
| Philippines | 0.775708 | -0.02037 | -0.01027 | 0.09049 | 0.054643 | -0.05799 | 0.014982 |
| Poland | 0.567924 | -0.0314 | 0.027651 | 0.03868 | 0.059136 | -0.01492 | 0.002622 |
| Romania | 0.65206 | -0.0076 | 0.083037 | -0.09001 | 0.183811 | -0.10491 | -0.01381 |
| Russia | 0.633429 | -0.01565 | -0.01301 | -0.03643 | -0.04199 | -0.09117 | -0.01904 |
| Rwanda | 0.59694 | -0.01793 | 0.00638 | -0.03023 | 0.018735 | -0.02137 | -0.08843 |
| Singapore | 0.620767 | 0.003088 | 0.028053 | -0.07294 | 0.122129 | -0.04423 | -0.01251 |
| Slovenia | 0.46464 | -0.01094 | 0.077195 | -0.05861 | 0.135055 | 0.003483 | -0.04056 |
| South Africa | 0.532036 | 0.01558 | 0.135891 | -0.07028 | 0.151689 | 0.010542 | -0.03563 |
| Zimbabwe | 0.585491 | -0.02354 | -0.03643 | -0.02994 | 0.058367 | -0.09031 | 0.014917 |
| Spain | 0.378936 | -0.00418 | 0.137876 | -0.10861 | 0.081728 | 0.033872 | -0.15984 |
| Sweden | 0.120131 | 0.018061 | 0.10649 | 0.015189 | 0.153677 | 0.038038 | -0.00486 |
| Thailand | 0.569339 | 0.037749 | 0.175806 | 0.174554 | 0.165117 | 0.002451 | 0.008463 |
| Trinidad and Tobago | 0.594486 | 0.000249 | 0.016013 | 0.020496 | 0.064359 | -0.03618 | -0.06825 |
| Turkey | 0.708226 | -0.06001 | -0.03273 | 0.154476 | -0.02756 | -0.02595 | -0.12548 |
| Ukraine | 0.627163 | -0.04749 | 0.04268 | 0.007969 | 0.024028 | -0.07353 | -0.04129 |
| Egypt | 0.893842 | -0.05003 | 0.019656 | 0.04663 | -0.00615 | -0.0221 | -0.06637 |
| United States | 0.414887 | 0.016065 | 0.092733 | -0.07296 | 0.057619 | 0.021587 | -0.01265 |
| Uruguay | 0.507965 | -0.0261 | -0.0221 | 0.032994 | 0.047227 | -0.00721 | -0.02309 |
| Yemen | 0.86783 | -0.01495 | -0.02235 | 0.205205 | 0.037438 | -0.0694 | -0.04784 |

Table S8

Estimated intercepts and slopes for social conservatism.

| countryname | Intercept | Surveillance Threat | Violence Threat | Economic Threat | Poverty Threat | Neighborhood Threat | Police Threat |
| --- | --- | --- | --- | --- | --- | --- | --- |
| Algeria | 0.858175 | -0.00492 | -0.0078 | 0.060604 | -0.01556 | -0.0519 | -0.0155 |
| Azerbaijan | 0.878654 | -0.02299 | -0.02433 | -0.0104 | 0.027597 | -0.1372 | -0.03162 |
| Argentina | 0.566573 | 0.017477 | 0.059403 | -0.03039 | 0.129558 | 0.004969 | -0.14458 |
| Australia | 0.405361 | 0.013701 | 0.179112 | -0.11925 | 0.1346 | 0.182462 | -0.08141 |
| Armenia | 0.906814 | 0.012318 | 0.013039 | -0.02207 | 0.023381 | -0.05488 | -0.01237 |
| Brazil | 0.728872 | -0.01484 | 0.0633 | -0.02566 | 0.030835 | -0.00723 | -0.06786 |
| Belarus | 0.785367 | -0.02646 | 0.158356 | -0.06442 | 0.031011 | -0.01902 | -0.01177 |
| Chile | 0.608709 | -0.03252 | 0.162794 | -0.02227 | 0.087669 | 0.046661 | 0.039203 |
| China | 0.832806 | -0.06771 | 0.098571 | -0.026 | -0.0556 | -0.09806 | -0.04428 |
| Taiwan | 0.650876 | 0.004235 | 0.140445 | -0.06149 | 0.106245 | 0.047047 | -0.05892 |
| Cyprus (G) | 0.679487 | 0.012633 | 0.092984 | -0.08937 | 0.076896 | 0.028386 | -0.0695 |
| Ecuador | 0.831592 | 0.010803 | -0.00173 | -0.02958 | -0.0106 | 0.016079 | -0.06776 |
| Estonia | 0.65531 | -0.00591 | 0.142147 | -0.06106 | 0.029715 | 0.037361 | -0.09933 |
| Georgia | 0.93833 | 0.000988 | 0.074213 | 0.007384 | -0.03344 | -0.04227 | -0.05441 |
| Palestine | 0.901167 | -0.01031 | -0.0146 | -0.01288 | 0.004804 | -0.07121 | -0.02886 |
| Germany | 0.496574 | -0.01951 | 0.004762 | -0.00055 | 0.105124 | 0.110249 | 0.027104 |
| Ghana | 0.93305 | 0.032588 | -0.06318 | 0.007834 | -0.01748 | 0.01834 | -0.00651 |
| Haiti | 0.70938 | -0.04789 | 0.009634 | -0.02869 | 0.041515 | -0.03549 | 0.035465 |
| Hong Kong | 0.699397 | -0.05236 | 0.143285 | -0.14801 | 0.068949 | -0.07494 | -0.03591 |
| India | 0.932507 | -0.00133 | -0.03564 | 0.046937 | -0.08925 | -0.07954 | -0.13088 |
| Iraq | 0.874774 | 0.000987 | 0.172336 | -0.00822 | -0.02435 | 0.029903 | -0.08769 |
| Japan | 0.560827 | 0.017926 | 0.149011 | -0.10723 | 0.156977 | 0.062298 | -0.05879 |
| Kazakhstan | 0.799959 | 0.041103 | 0.031567 | 0.037208 | -0.02727 | 0.001734 | -0.238 |
| Jordan | 0.931487 | -0.02343 | 0.021385 | -0.00413 | -0.02907 | -0.07719 | -0.0086 |
| South Korea | 0.717648 | -0.01037 | 0.07877 | -0.05485 | 0.090723 | -0.0941 | -0.06586 |
| Kyrgyzstan | 0.883408 | -0.03877 | 0.054096 | -0.02163 | -0.02951 | -0.1182 | -0.07112 |
| Lebanon | 0.784332 | -0.04239 | 0.208087 | 0.05219 | -0.12778 | -0.01164 | -0.1943 |
| Libya | 0.916701 | -0.00521 | -0.02393 | 0.083449 | -0.04607 | -0.01842 | -0.01876 |
| Malaysia | 0.865378 | -0.02348 | 0.24589 | -0.07684 | -0.01753 | -0.07062 | -0.06846 |
| Mexico | 0.718136 | -0.00987 | 0.07641 | -0.04522 | 0.04513 | -0.0323 | -0.0473 |
| Morocco | 0.946564 | 0.016866 | 0.023336 | -0.0363 | -0.00144 | -0.05784 | -0.04469 |
| Netherlands | 0.284107 | 0.009799 | 0.325105 | -0.11303 | 0.225035 | 0.199763 | -0.04808 |
| New Zealand | 0.488277 | 0.046702 | 0.230457 | -0.10524 | 0.146208 | 0.162867 | -0.13451 |
| Nigeria | 0.875753 | -0.02693 | 0.096129 | -0.02656 | -0.04748 | -0.01144 | -0.03962 |
| Pakistan | 0.936331 | -0.01389 | 0.143879 | 0.031836 | -0.03631 | -0.06134 | -0.02251 |
| Peru | 0.77202 | -0.03344 | 0.042457 | -0.01432 | -0.00617 | -0.02594 | -0.085 |
| Philippines | 0.686599 | -0.07797 | 0.099057 | -0.01801 | -0.01755 | 0.020708 | 0.041528 |
| Poland | 0.719029 | -0.04274 | 0.077968 | -0.06467 | 0.054058 | -0.03586 | 0.032596 |
| Romania | 0.815816 | 0.004575 | 0.076843 | -0.07028 | 0.046652 | -0.02016 | -0.07187 |
| Russia | 0.718052 | -0.003 | 0.091374 | -0.01267 | -0.00668 | 0.045835 | -0.06113 |
| Rwanda | 0.901753 | -0.00183 | 0.266539 | -0.01021 | -0.00361 | -0.02774 | 0.000156 |
| Singapore | 0.703492 | -0.03922 | 0.090319 | -0.04705 | 0.019436 | -0.0146 | 0.001691 |
| Slovenia | 0.424743 | 0.027507 | 0.16636 | -0.10925 | 0.115555 | 0.174187 | -0.21419 |
| South Africa | 0.619421 | -0.0691 | -0.03608 | 0.167305 | -0.18074 | 0.16543 | -0.20518 |
| Zimbabwe | 0.890768 | 0.022069 | 0.026222 | 0.028894 | -0.04298 | 0.012166 | -0.11249 |
| Spain | 0.415541 | -0.05327 | 0.126811 | -0.21045 | 0.106596 | 0.092452 | -0.03191 |
| Sweden | 0.201178 | -0.03524 | 0.150721 | -0.03144 | 0.172543 | 0.227068 | -0.01131 |
| Thailand | 0.859213 | -0.00996 | 0.109 | -0.01453 | 0.031861 | -0.16075 | -0.09804 |
| Trinidad and Tobago | 0.896164 | -0.02083 | 0.029602 | -0.04351 | -0.01782 | -0.05746 | -0.00937 |
| Tunisia | 0.929176 | 0.020262 | -0.01111 | 0.002948 | -0.00452 | -0.03355 | -0.03054 |
| Turkey | 0.884725 | 0.040961 | -0.05115 | -0.01451 | 0.005769 | -0.0153 | -0.11752 |
| Ukraine | 0.77616 | -0.01968 | 0.166093 | -0.05429 | 0.005841 | -0.05031 | -0.09081 |
| United States | 0.544726 | 0.032303 | 0.131007 | -0.07378 | 0.08312 | 0.145613 | -0.13299 |
| Uruguay | 0.542452 | 0.006741 | 0.12442 | 0.019344 | 0.060158 | 0.120768 | -0.05085 |
| Yemen | 0.91562 | -0.00724 | 0.228203 | -0.0417 | -0.00433 | -0.08268 | -0.05221 |

Table S9

Estimated intercepts and slopes for ideology.

| country | Intercept | Surveillance Threat | Violence Threat | Economic Threat | Poverty Threat | Neighborhood Threat | Police Threat |
| --- | --- | --- | --- | --- | --- | --- | --- |
| Algeria | 0.553215 | -0.04529 | -0.00141 | -0.06503 | 0.029087 | -0.07394 | -0.05347 |
| Azerbaijan | 0.546618 | 0.020612 | 0.01234 | 0.108824 | -0.04031 | 0.063168 | 0.021651 |
| Argentina | 0.485489 | -0.01864 | 0.100773 | 0.014813 | -0.02121 | 0.007156 | -0.05124 |
| Australia | 0.477716 | -0.03825 | 0.083915 | -0.06644 | -0.0507 | 0.062906 | -0.09347 |
| Armenia | 0.521718 | -0.02049 | 0.050551 | 0.047408 | -0.0278 | -0.05913 | -0.00496 |
| Brazil | 0.47935 | -0.02793 | 0.045653 | -0.0167 | 0.089198 | -0.07611 | -0.05593 |
| Belarus | 0.472947 | -0.01065 | 0.030826 | 0.001188 | -0.01607 | -0.02651 | -0.00468 |
| Chile | 0.451517 | -0.04796 | 0.017816 | -0.02718 | -0.03868 | -0.0046 | -0.04912 |
| Taiwan | 0.400353 | -0.04939 | 0.040607 | -0.04887 | 0.027341 | 0.020905 | -0.09625 |
| Cyprus (G) | 0.464006 | -0.04036 | -0.03582 | -0.01179 | -0.00813 | -0.06 | -0.01175 |
| Ecuador | 0.507111 | 0.025282 | -0.02546 | -0.05577 | 0.004112 | -0.05724 | 0.058982 |
| Estonia | 0.48533 | -0.04338 | -0.0014 | -0.00191 | -0.05839 | -0.08019 | -0.00054 |
| Georgia | 0.504752 | -0.04338 | 0.0863 | 0.012841 | -0.00261 | -0.04615 | -0.06591 |
| Palestine | 0.554734 | -0.02604 | 0.008212 | -0.01038 | 0.007547 | -0.03234 | -0.03276 |
| Germany | 0.43231 | -0.01275 | 0.011935 | 0.013483 | 0.014129 | -0.02227 | -0.00943 |
| Ghana | 0.503814 | -0.00262 | 0.034549 | 0.122114 | -0.05919 | 0.035174 | 0.005211 |
| Haiti | 0.189829 | -0.01196 | -0.04636 | -0.03443 | 0.01209 | 0.044364 | -0.02472 |
| Hong Kong | 0.491342 | 0.006908 | -0.02821 | -0.02407 | -0.04456 | -0.01103 | 0.035464 |
| India | 0.514281 | 0.046372 | 0.011591 | 0.013193 | 0.001943 | -0.0366 | 0.069881 |
| Iraq | 0.583397 | -0.03922 | 0.115412 | 0.043786 | 0.130375 | -0.00629 | -0.13796 |
| Japan | 0.510279 | -0.035 | 0.119571 | -0.03872 | 0.018368 | -0.05797 | -0.06905 |
| Kazakhstan | 0.570121 | 0.044458 | -0.10018 | 0.081061 | 0.076892 | -0.02695 | 0.082789 |
| South Korea | 0.472908 | -0.02648 | 0.000101 | -0.03607 | 0.021634 | -0.1147 | -0.00273 |
| Kyrgyzstan | 0.596377 | 0.010351 | -0.00954 | 0.137788 | -0.01667 | -0.03405 | 0.057008 |
| Lebanon | 0.598679 | 0.007364 | -0.06657 | -0.05508 | 0.009887 | -0.02657 | 0.030052 |
| Libya | 0.557995 | 0.008752 | -0.01456 | -0.02511 | -0.03441 | -0.11544 | 0.075843 |
| Malaysia | 0.626575 | -0.04244 | 0.046789 | 0.090363 | 0.031395 | -0.21741 | 0.007853 |
| Mexico | 0.580955 | -0.02202 | 0.060282 | 0.019862 | -0.03685 | -0.05932 | -0.00554 |
| Morocco | 0.503677 | -0.01852 | 0.069752 | 0.019851 | -0.059 | -0.02584 | -0.01227 |
| Netherlands | 0.515658 | -0.01409 | 0.031941 | -0.03457 | -0.02368 | 0.000275 | -0.02205 |
| New Zealand | 0.524942 | -0.02918 | 0.070941 | -0.04068 | -0.02217 | 0.022884 | -0.06867 |
| Nigeria | 0.511614 | 0.031877 | 0.032473 | 0.026133 | -0.2023 | 0.000374 | 0.118221 |
| Pakistan | 0.704165 | -0.00206 | 0.162433 | 0.117301 | -0.04088 | 0.013967 | -0.01867 |
| Peru | 0.506916 | 0.025045 | -0.00318 | 0.057454 | -0.01062 | -0.00551 | 0.050514 |
| Philippines | 0.626894 | 0.00357 | 0.086852 | 0.028325 | 0.064273 | -0.02419 | -0.03123 |
| Poland | 0.497012 | 0.014269 | 0.013729 | -0.02757 | -0.05198 | -0.0095 | 0.031966 |
| Romania | 0.518961 | 0.035129 | -0.01481 | 0.015487 | -0.01102 | -0.05361 | 0.082524 |
| Russia | 0.498383 | 0.025471 | -0.03346 | 0.106849 | 0.026701 | 0.007831 | 0.041102 |
| Rwanda | 0.494043 | 0.00646 | -0.01243 | 0.003181 | -0.0192 | 0.086624 | -0.00861 |
| Slovenia | 0.44939 | -0.03952 | 0.04803 | 0.009483 | 0.02523 | 0.012004 | -0.08089 |
| South Africa | 0.58955 | 0.022032 | 0.059987 | -0.02187 | -0.03604 | -0.04494 | 0.040079 |
| Zimbabwe | 0.47202 | -0.06113 | -0.02722 | 0.009552 | -0.00381 | -0.11285 | -0.0208 |
| Spain | 0.424367 | -0.0279 | 0.003077 | -0.02254 | -0.00603 | 0.068343 | -0.06457 |
| Sweden | 0.499674 | -0.0181 | 0.127227 | -0.11076 | -0.10395 | 0.011221 | -0.04468 |
| Thailand | 0.544794 | 0.017783 | 0.009224 | 0.077725 | 0.014008 | -0.02444 | 0.033562 |
| Trinidad and Tobago | 0.603271 | -0.04134 | 0.067887 | 0.00578 | -0.01032 | -0.05976 | -0.05179 |
| Tunisia | 0.509568 | -0.00707 | -0.03048 | 0.094959 | 0.033353 | -0.04096 | 0.01524 |
| Turkey | 0.592446 | -0.06376 | 0.054295 | 0.017773 | 0.006194 | -0.06356 | -0.07269 |
| Ukraine | 0.510564 | 0.027955 | -0.00331 | 0.063701 | -0.00234 | -0.03826 | 0.059352 |
| Egypt | 0.568626 | 0.006329 | -0.06032 | -0.04625 | -0.10579 | -0.07465 | 0.090308 |
| United States | 0.531951 | -0.03379 | 0.125173 | -0.01628 | -0.05482 | -0.03166 | -0.05534 |
| Uruguay | 0.401536 | 0.000528 | 0.043419 | -0.08715 | -0.03483 | -0.03979 | -0.0034 |
| Yemen | 0.519351 | -0.0432 | 0.134401 | -0.12939 | -0.06198 | -0.05415 | -0.06498 |


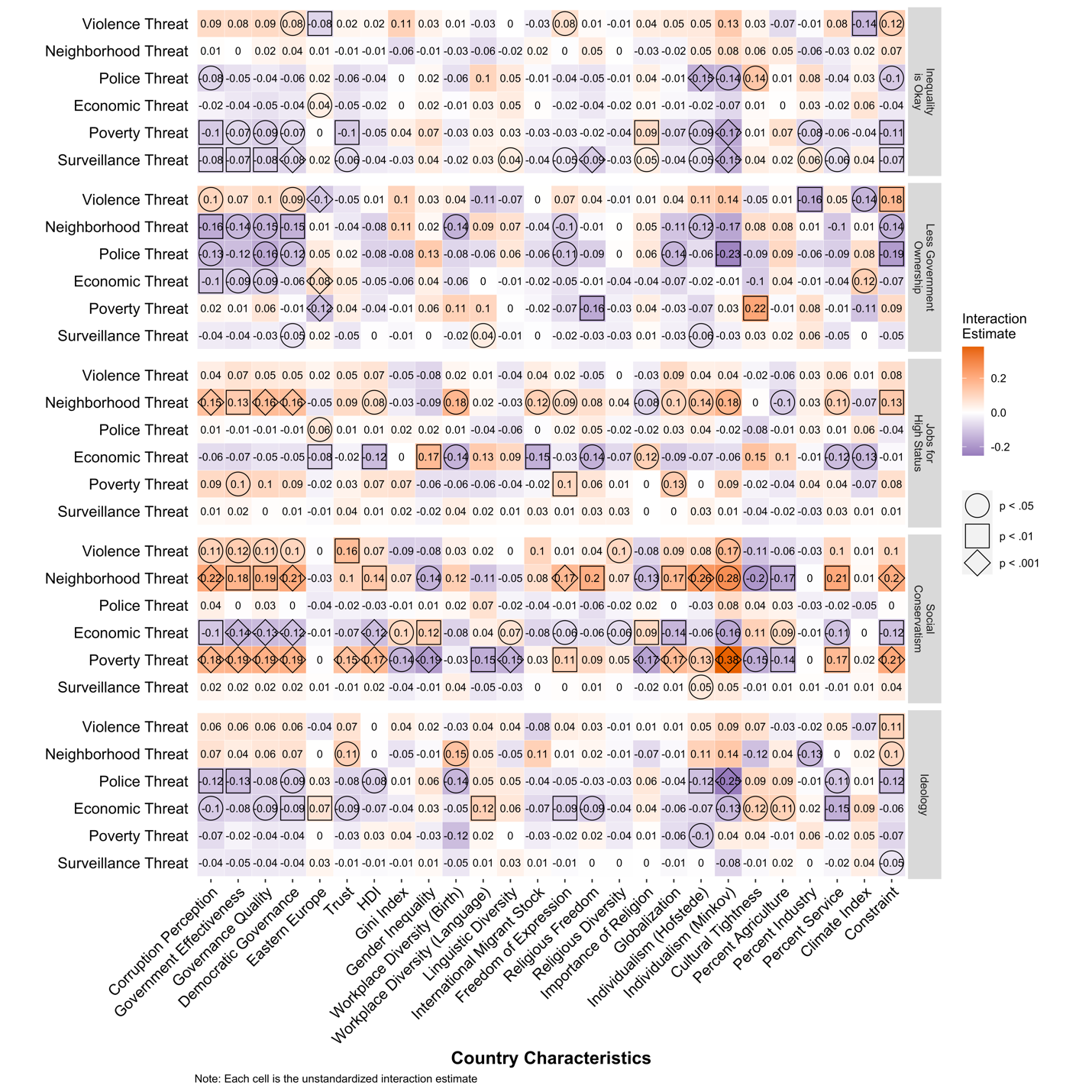


**Figure S3. Estimates for the interaction between country characteristics (x-axis) and threats (y-axis) for the five measures of political beliefs (row chunks). Estimates that are significant without any alpha correction are highlighted with geometric shapes.**

## Digging Deeper into Government Effectiveness^^[[1]](#footnote-1)^^

We had anticipated that threats may be more strongly associated with political beliefs that give governments power, such as left-wing economic beliefs, when a country has a well-functioning government. To the extent there was evidence for this, it was only the case for surveillance threat when predicting the beliefs that inequality is okay. When countries were less corrupt (Figure S4), more democratic (Figure S5), and were higher quality (Figure S7) surveillance threats were associated with the more left-wing position; however, when countries were more corrupt, less democratic, and less effective surveillance threats were associated with the more right-wing position. This same type of relationship did not emerge for any of the other threats nor for any of the other economic beliefs. Overall, our expectation was not supported.

There were more significant interactions when predicting cultural beliefs (see Figures S4-S7). As one example, in more democratic countries, neighborhood threat was associated with both jobs for high status and social conservatism beliefs, but this association was reversed in less democratic countries (Figure S5). At the same time, economic threat was associated with less social conservatism in democratic countries and it was unrelated to social conservatism in less democratic countries.


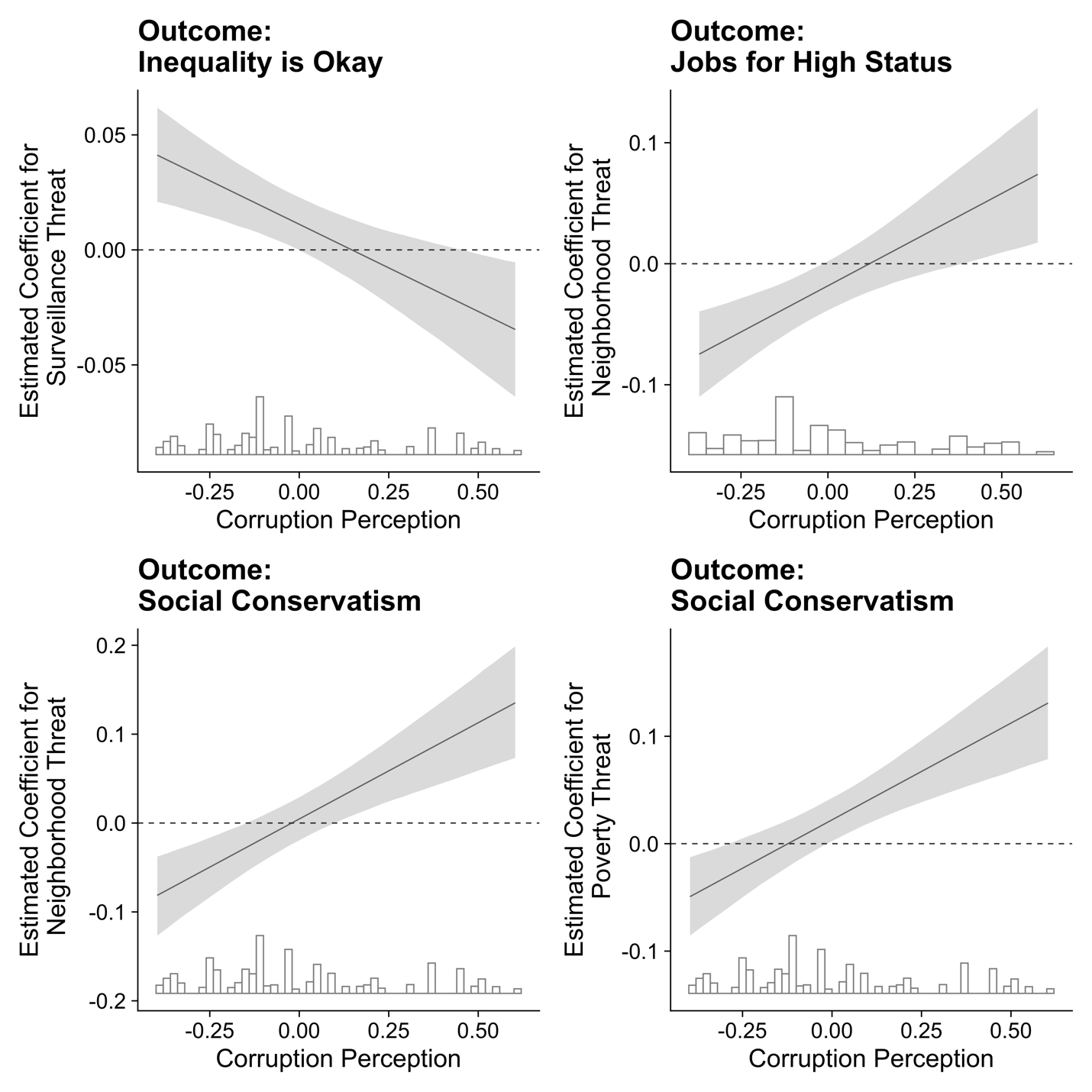


**Figure S4. Coefficient of the estimated simple slope (y-axis) at different levels of corruption perception (x-axis, higher scores = less corruption). Gray shading highlights 95% confidence intervals. Zero on the y-axis indicates a non-significant association between threat and the outcome.**


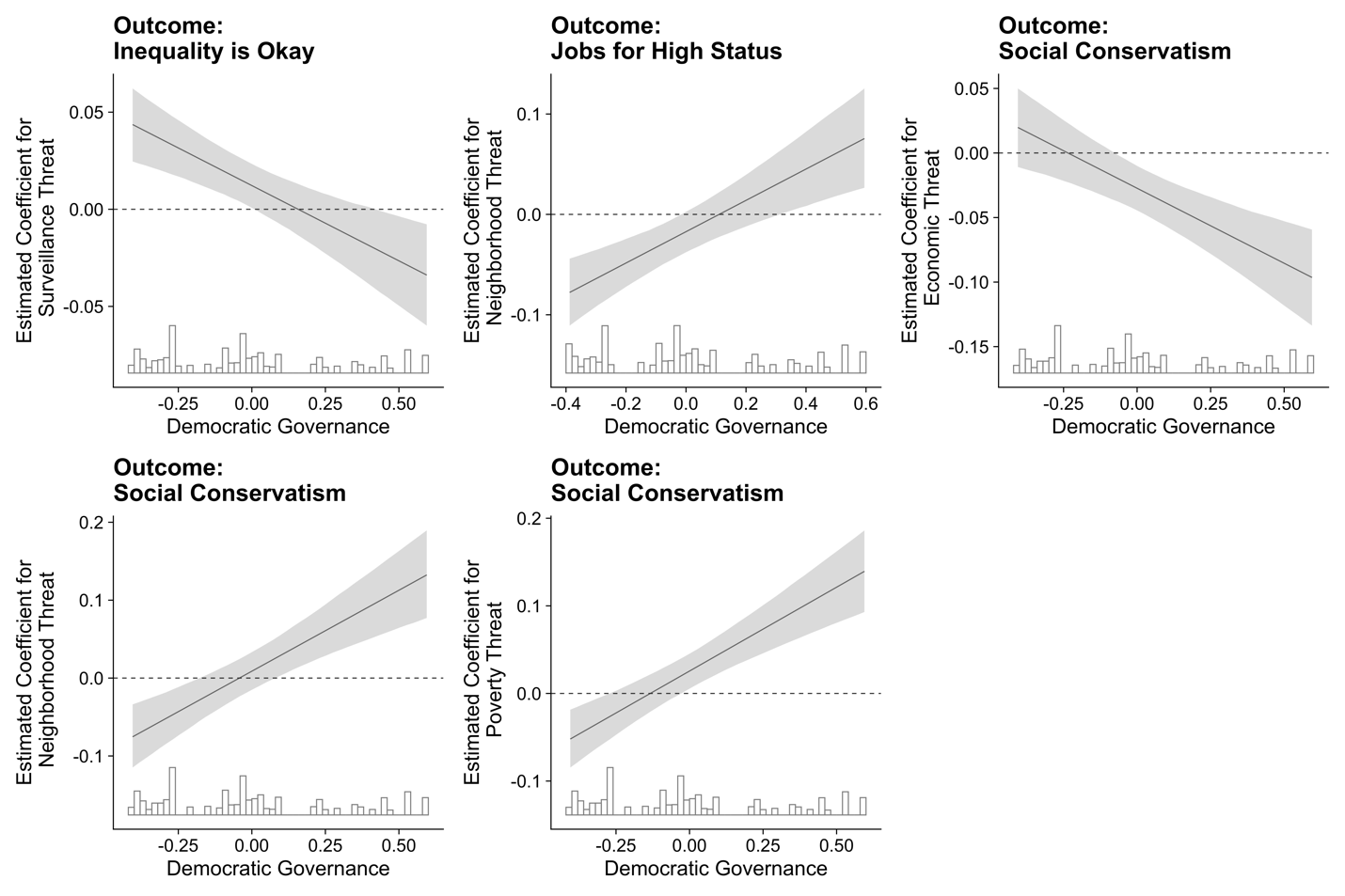


**Figure S5.** **Coefficient of the estimated simple slope (y-axis) at different levels of democratic governance (x-axis). Gray shading highlights 95% confidence intervals. Zero on the y-axis indicates a non-significant association between threat and the outcome.**


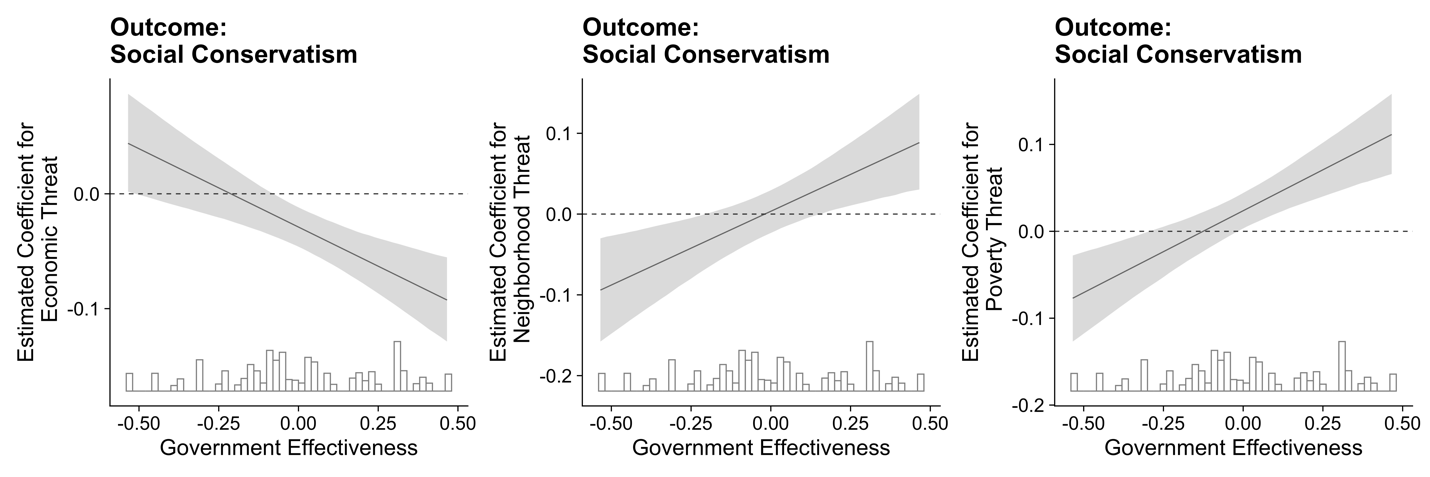


**Figure S6.** **Coefficient of the estimated simple slope (y-axis) at different levels of government effectiveness (x-axis). Gray shading highlights 95% confidence intervals. Zero on the y-axis indicates a non-significant association between threat and the outcome.**


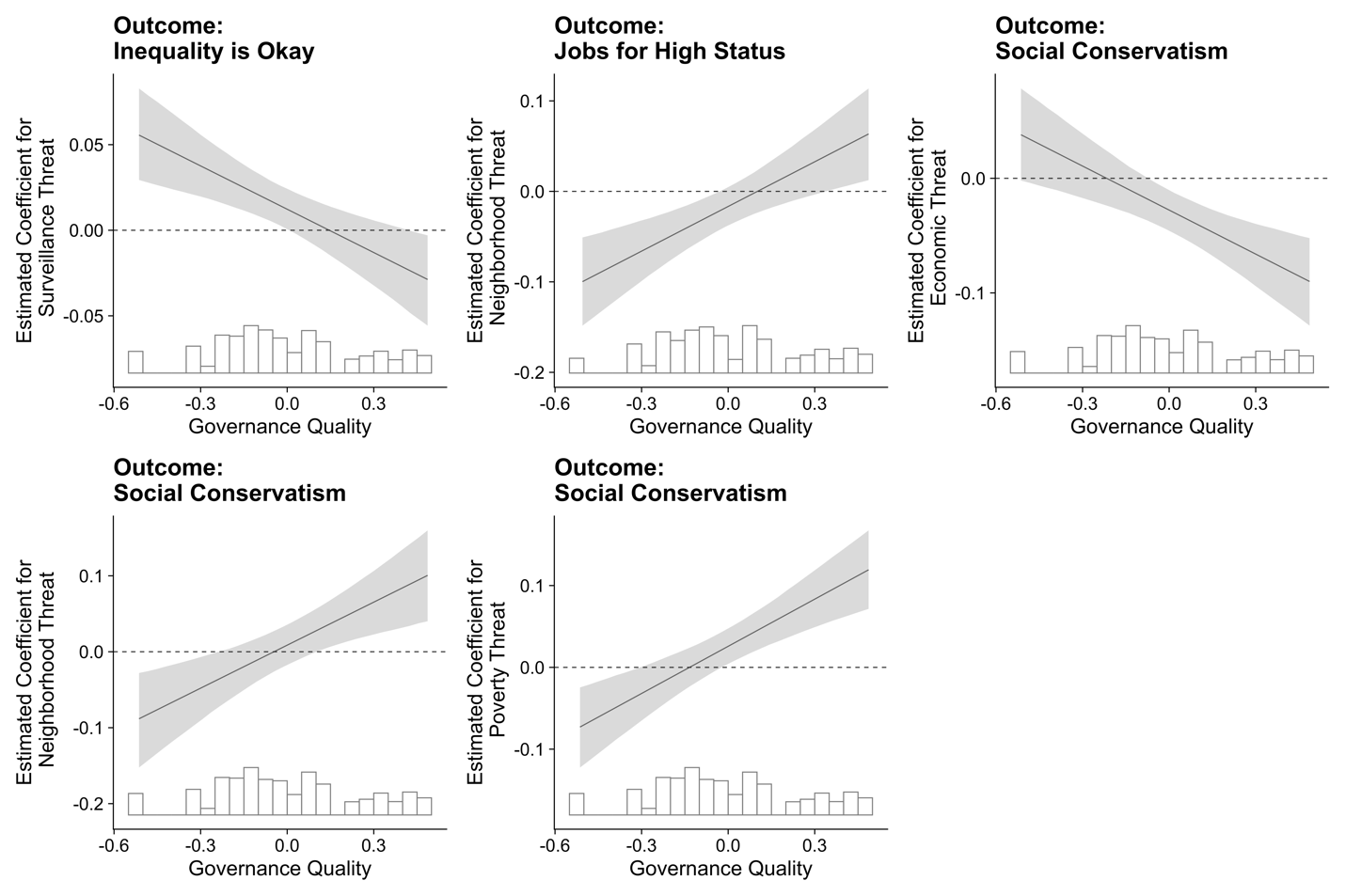


**Figure S7. Coefficient of the estimated simple slope (y-axis) at different levels of governance quality (x-axis). Gray shading highlights 95% confidence intervals. Zero on the y-axis indicates a non-significant association between threat and the outcome.**

## Digging Deeper into ‘Located in former Eastern Bloc’

We expected that threats may be more likely associated with left-wing economic political beliefs in countries from the former Eastern Bloc. This was the case for both violence threat and poverty threat when predicting less government ownership (Figure S8). In both cases, the threat coefficient was more negative in Eastern Bloc countries than in non-Eastern Bloc countries. However, for economic threat this pattern was reverse. Economic threat was associated with more right-wing beliefs about government ownership in countries from the former Eastern Bloc. Therefore, there is some evidence for our prediction, but it does not extend to most of threats, sometimes is in the opposite direction, and only applies to one of the possible measures of economic beliefs.


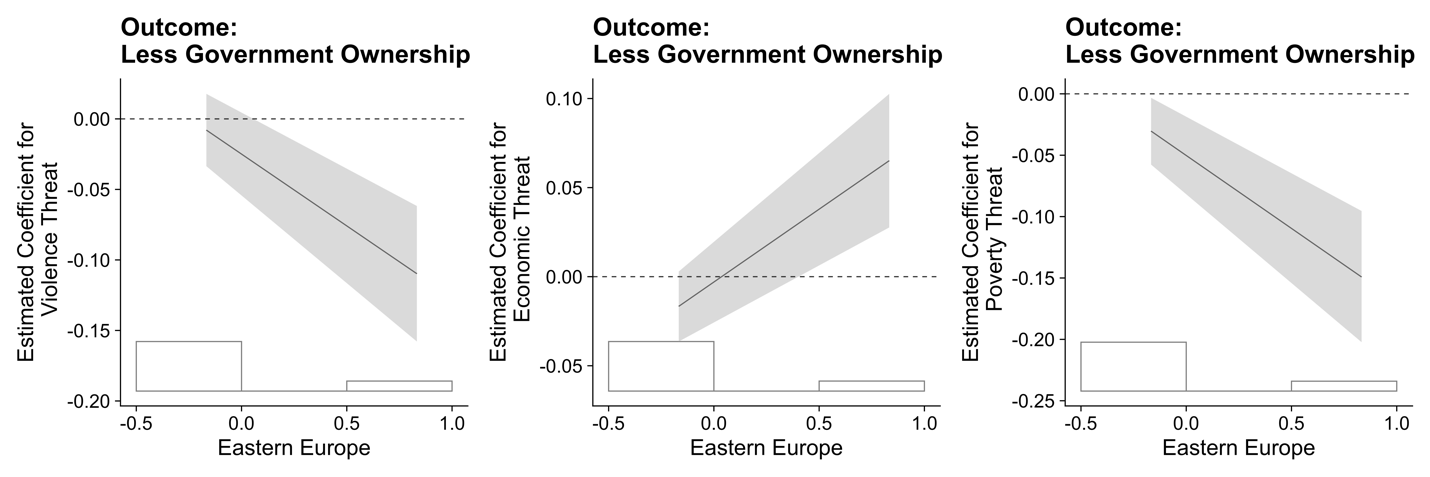


**Figure S8. Coefficient of the estimated simple slope (y-axis) for countries in Eastern Europe (.5) or not (-.5) (x-axis). Gray shading highlights 95% confidence intervals. Zero on the y-axis indicates a non-significant association between threat and the outcome.**

## Digging Deeper into ‘Threatening Economic Conditions’

We had anticipated that threats may be more weakly associated with right-wing political views when economic conditions are poor. In two cases (middle and right panel, Figure S9), this seemed to be the case. In countries with higher scores on the human development index, poverty threat is positively associated with social conservatism. However, in countries with lower scores, poverty threat is negatively associated with social conservatism. In countries with lower scores on gender inequality (i.e., relatively good economic conditions for women), poverty treat was positively associated with social conservatism. In contrast, in countries with higher gender inequality (i.e., relatively bad economic conditions for women), poverty treat was negatively associated with social conservatism. In one other case, the effect was opposite of the prediction (see left panel Figure S9). There were no other significant interactions with the variables associated with economic conditions, suggesting that our expectations were not confirmed.


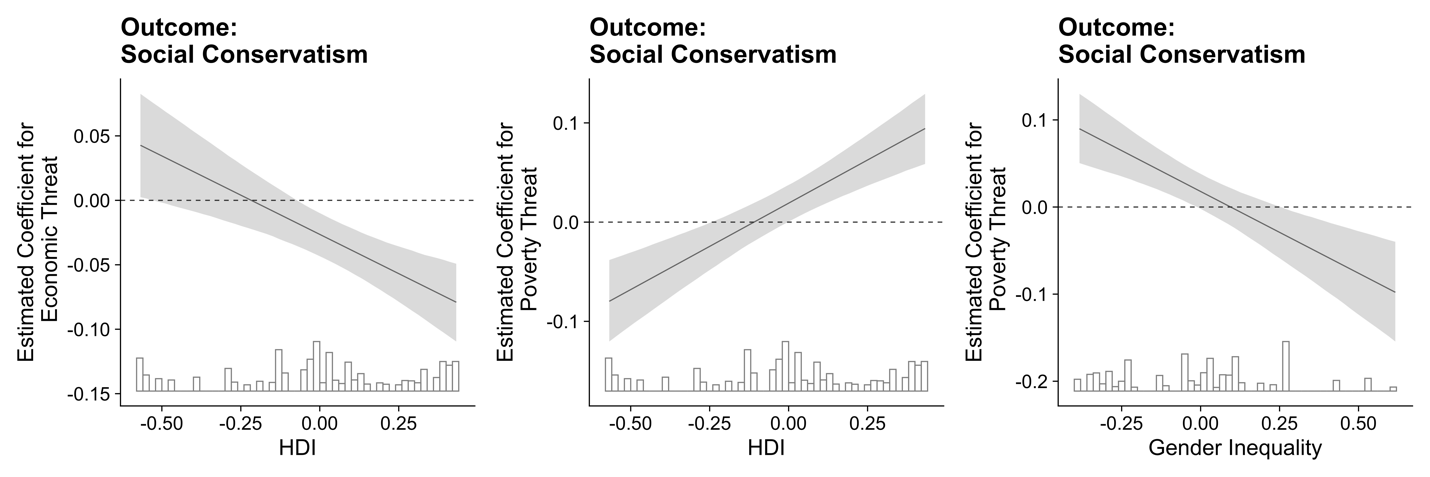


**Figure S9. Coefficient of the estimated simple slope (y-axis) at different levels of the Human Development Index or Gender Inequality (x-axis). Gray shading highlights 95% confidence intervals. Zero on the y-axis indicates a non-significant association between threat and the outcome.**

## Digger Deeper into ‘Ideological Constraint’

We had anticipated that threats may be more likely to be associated with right-wing economic beliefs in countries with more ideological constraint. This was the case for violence threat predicting less government ownership (Figure S10). It was not the case for any other threat or economic belief. There were similarly shaped interactions for both neighborhood threat and poverty threat when predicting social conservatism. Overall, it does not appear that the expectations were supported.


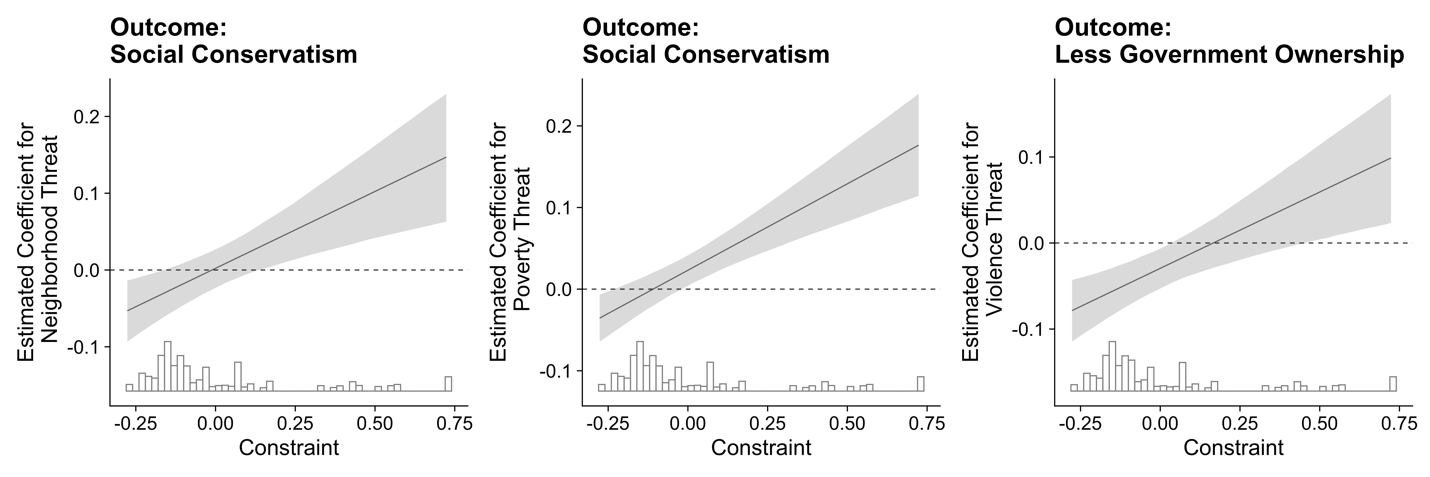


**Figure S10. Coefficient of the estimated simple slope (y-axis) at different levels of the constraint (x-axis). Gray shading highlights 95% confidence intervals. Zero on the y-axis indicates a non-significant association between threat and the outcome.**

# Plots of the Remaining Significant Interactions


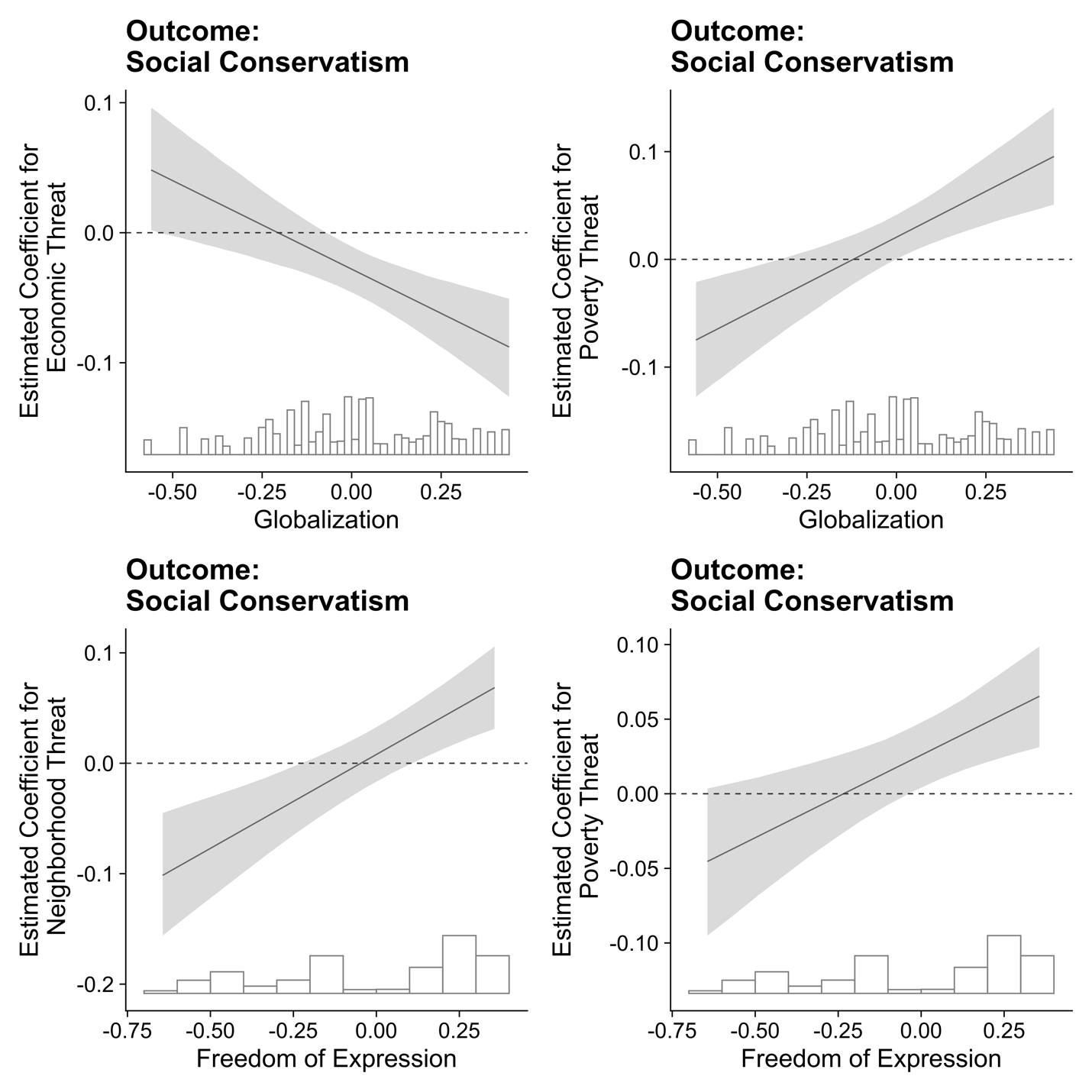


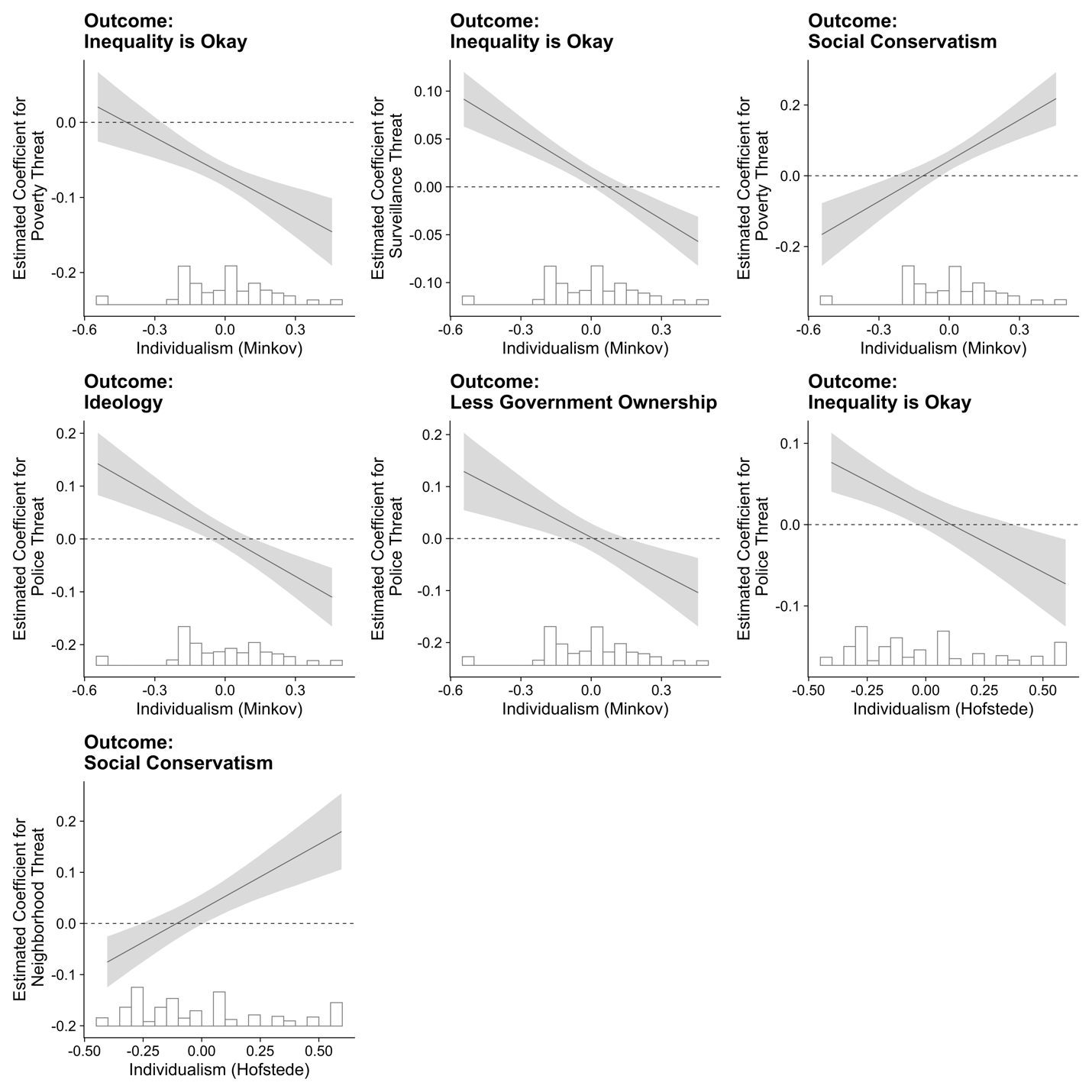


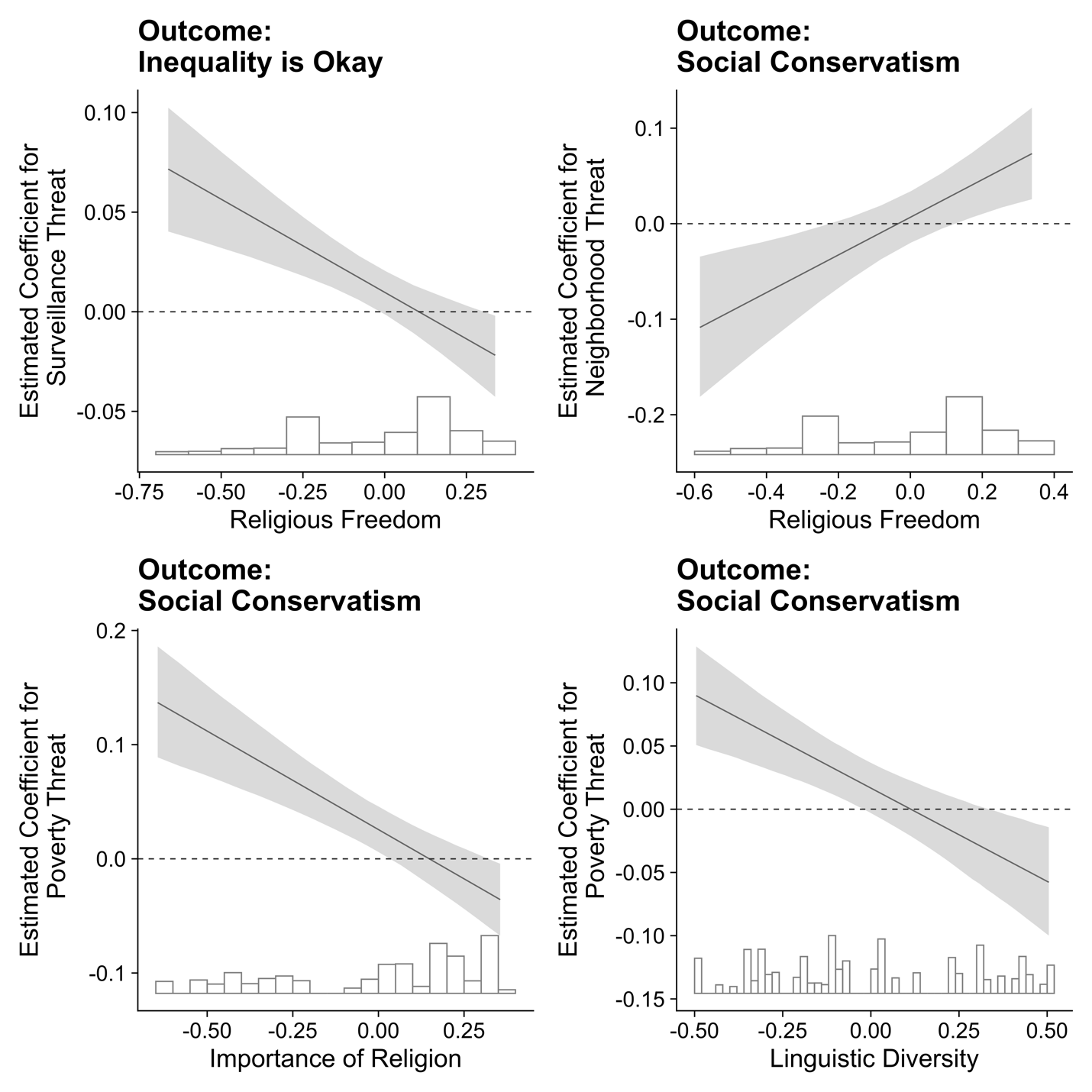


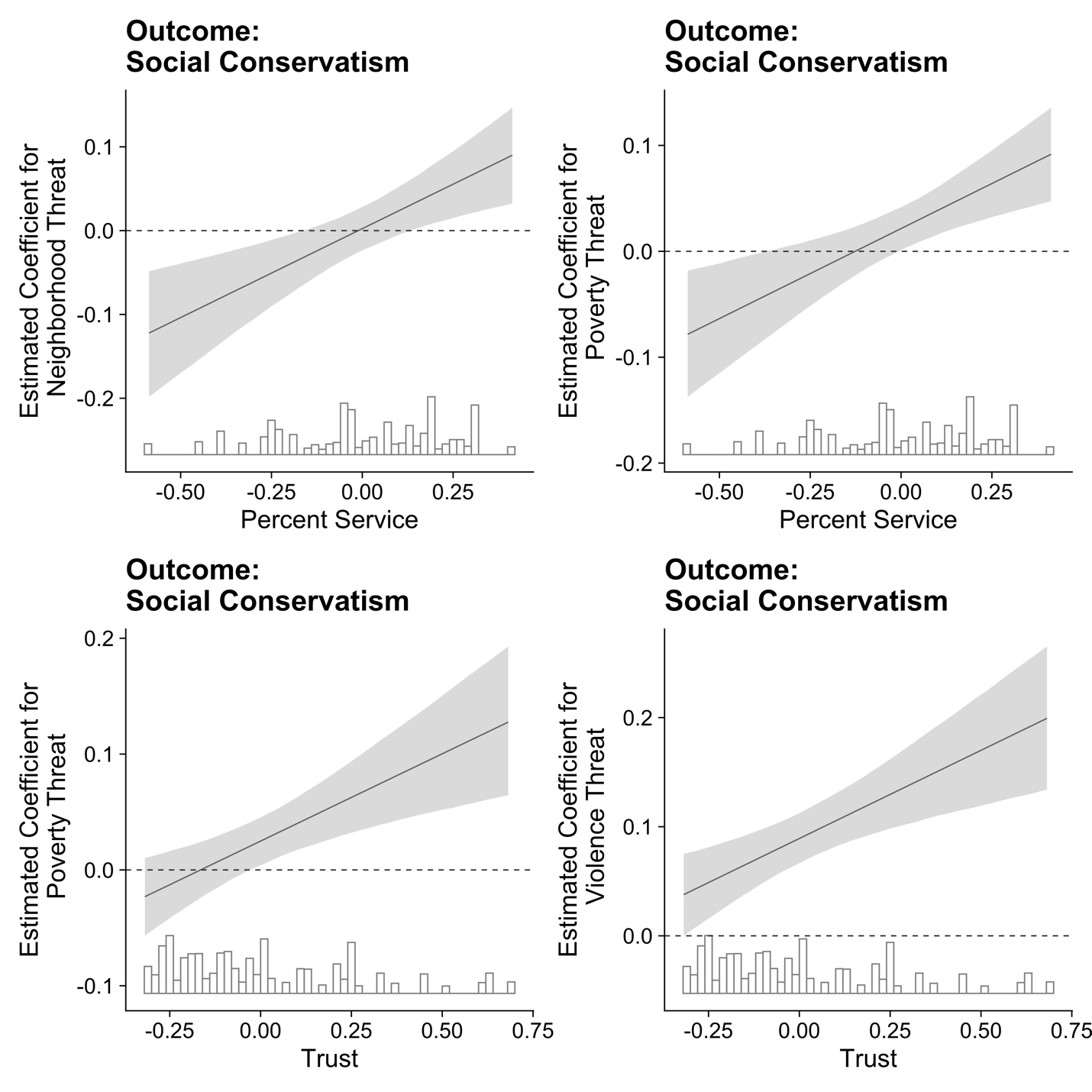


1. All digging deeper sections discuss significant interactions in Figure 7 from the main text. [↑](#footnote-ref-1)
